# Mechanical overload-induced release of extracellular mitochondrial particles from tendon cells leads to inflammation in tendinopathy

**DOI:** 10.1038/s12276-024-01183-5

**Published:** 2024-03-01

**Authors:** Ziming Chen, Mengyuan Li, Peilin Chen, Andrew Tai, Jiayue Li, Euphemie Landao Bassonga, Junjie Gao, Delin Liu, David Wood, Brendan F. Kennedy, Qiujian Zheng, Ming H. Zheng

**Affiliations:** 1 Centre for Orthopaedic Research, School of Surgery, The University of Western Australia, Nedlands, WA 6009 Australia; 2 Department of Joint Osteopathy and Traumatology, Guangdong Provincial People’s Hospital (Guangdong Academy of Medical Sciences), Southern Medical University, Guangdong, China; 3 School of Medicine, Monash University, Clayton, VIC VIC 3800 Australia; 4 Perron Institute for Neurological and Translational Science, Nedlands, WA 6009 Australia; 5 BRITElab, Harry Perkins Institute of Medical Research, QEII Medical Centre, Nedlands, and Centre for Medical Research, The University of Western Australia, Perth, WA 6009 Australia; 6 Department of Electrical, Electronic and Computer Engineering, School of Engineering, The University of Western Australia, Nedlands, WA 6009 Australia; 7 Australian Research Council Centre for Personalised Therapeutics Technologies, Melbourne, VIC Australia; 8 Department of Orthopaedics, Shanghai Jiao Tong University Affiliated Shanghai Sixth People’s Hospital, Shanghai, 200233 China; 9 Institute of Physics, Faculty of Physics, Astronomy and Informatics, Nicolaus Copernicus University in Toruń, Grudziadzka 5, 87-100, Torun, Poland

**Keywords:** Chronic inflammation, Experimental models of disease, Trauma, Energy metabolism

## Abstract

Tendinopathy is one of the most common musculoskeletal diseases, and mechanical overload is considered its primary cause. However, the underlying mechanism through which mechanical overload induces tendinopathy has not been determined. In this study, we identified for the first time that tendon cells can release extracellular mitochondria (ExtraMito) particles, a subtype of medium extracellular particles (mEPs), into the environment through a process regulated by mechanical loading. RNA sequencing systematically revealed that oxygen-related reactions, extracellular particles, and inflammation were present in diseased human tendons, suggesting that these factors play a role in the pathogenesis of tendinopathy. We simulated the disease condition by imposing a 9% strain overload on three-dimensional mouse tendon constructs in our cyclic uniaxial stretching bioreactor. The three-dimensional mouse tendon constructs under normal loading with 6% strain exhibited an extended mitochondrial network, as observed through live-cell confocal laser scanning microscopy. In contrast, mechanical overload led to a fragmented mitochondrial network. Our microscopic and immunoblot results demonstrated that mechanical loading induced tendon cells to release ExtraMito particles. Furthermore, we showed that mEPs released from tendon cells overloaded with a 9% strain (mEP_9%_) induced macrophage chemotaxis and increased the production of proinflammatory cytokines, including IL-6, CXCL1, and IL-18, from macrophages compared to mEP_0%_, mEP_3%_, and mEP_6%_. Partial depletion of the ExtraMito particles from mEP_9%_ by magnetic-activated cell sorting significantly reduced macrophage chemotaxis. N-acetyl-L-cysteine treatment preserved the mitochondrial network in overloaded tendon cells, diminishing overload-induced macrophage chemotaxis toward mEP_9%_. These findings revealed a novel mechanism of tendinopathy; in an overloaded environment, ExtraMito particles convey mechanical response signals from tendon cells to the immune microenvironment, culminating in tendinopathy.

## Mechanical overload unveils new culprit in tendinopathy: extracellular mitochondria

Tendons, essential tissues linking muscles to bones, can deteriorate due to repeated stress, causing tendinopathy—a painful condition affecting many. Despite usual treatments like painkillers and physical therapy, there’s a shortage of therapies addressing the primary causes of tendinopathy. They gathered tendon samples from patients, conducted RNA sequencing, and used a three-dimensional bioreactor to mimic tendon stress. This research, involving Minghao Zheng, aimed to bridge this knowledge gap by studying the cellular reactions at the molecular level in human tendons affected by tendinopathy. They discovered that stressed tendons release particles containing mitochondria, which then affect immune cells and can cause inflammation—a primary factor in tendinopathy. The study showed stressed tendons release particles that can initiate inflammation, suggesting a new understanding of the fundamental mechanisms of tendinopathy. The results could lead to innovative treatments targeting these cellular processes. This summary was initially drafted using artificial intelligence, then revised and fact-checked by the author.

## Introduction

The tendon primarily consists of type I collagen, which is stretched into parallel elastic fiber bundles, with tendon cells embedded within the matrix^1^. The main function of the tendon is to transmit the force generated from the muscles to the bones at the entheses, thus driving joint movements. Because of the uniqueness of the tendon’s physiological function and structural features, tendons undergo numerous repetitive stretches daily. This regular stress often leads to tendon degeneration, which gives rise to tendinopathy^2^.

Tendinopathy is one of the most common musculoskeletal diseases, with a 10.52 per 1000 person-years incidence of tendinopathy in the lower limb^2^ and a 2% to 3.8% incidence of tendinopathy in the upper limb for adults in the general population^3,4^. Tendinopathy seriously affects the quality of life of patients. Up to 5% of patients with upper limb tendinopathy have taken approximately 29 sick days from work^5^. Tendinopathy also poses a substantial economic burden. Absenteeism due to lateral epicondylitis was estimated to cost approximately £27 million annually in the United Kingdom alone^6^. Moreover, tendinopathy may further develop into tendon rupture, which leads to limb dysfunction and ultimately surgical intervention^7^.

While tendinopathy can be managed well with analgesics and physical therapy in most patients, effective treatments that directly address the underlying pathogenesis are still lacking^8^. One main reason is that the underlying pathological mechanism remains unclear. Multiple factors contribute to the pathogenesis of tendinopathy. Some studies have reported that tendinopathy is a process of extracellular matrix (ECM) degeneration and dysregulation^9,10^. Moreover, over the past decade, studies have reported that inflammation plays a role in the development of tendinopathy^11^. Tendon cells, as the residential stromal component, interact with immune cells from the infiltrating compartment and immune-sensing compartment, resulting in a complex cascade of inflammatory responses and subsequent matrix degradation^11^. Although the intrinsic mechanisms of tendinopathy remain a matter of debate, some theories propose that these pathological changes might work together in the development of tendinopathy^12,13^. Subcellular responses, such as lysosomal quality control, endoplasmic reticulum stress, and mitochondrial dynamics, have been found to affect both inflammation and tissue regeneration^14–16^. However, the subcellular responses in tendinopathy are largely unknown.

In this study, we performed RNA sequencing (RNA-seq) on human tendons with tendinopathy to systematically explore the cellular responses in tendinopathy at the molecular level. We used an in vitro three-dimensional (3D) uniaxial stretching bioreactor platform to closely mimic the in vivo mechanical stimulation of tendons. Based on data obtained from transcriptomic profiling, the 3D uniaxial stretching bioreactor platform, live-cell confocal laser scanning microscopy (CLSM), and magnetic-activated cell sorting (MACS) technology, we identified for the first time that extracellular mitochondria (ExtraMito), a novel subpopulation of tendon-derived medium extracellular particles (mEPs), play an essential role in passing mechanical information from tendon cells into the immune microenvironment. In this study, we explored new pathological mechanisms at the subcellular level that can potentially be used to address tendinopathy.

## Materials and methods

### Human tendon sample collection

The collection of human tendon samples for sequencing analysis was performed according to the protocols reviewed and approved by the Ethics Committee of Guangdong Provincial People’s Hospital (ethics approval number KY-Z-2020-586-02). The study was conducted based on the guidelines of the Declaration of Helsinki. Informed consent was obtained from the donors prior to tissue collection. The patient information remains confidential. Limited patient data, including sex and tendinopathy-related medical descriptions, were obtained. Unhealthy tendon tissues were surgically collected from 2 patients diagnosed with rotator cuff tendinopathy by magnetic resonance imaging. Healthy tendon tissues were collected from the hamstring tendons of 3 patients who underwent anterior cruciate ligament reconstruction.

### RNA sequencing, mapping, and analysis

Total RNA was extracted from human tendon samples by TRIzol reagent (Invitrogen, Waltham, United States). The RNA quality was determined by an Agilent 2200 instrument, and the samples were stored at −80 °C. RNA with an RNA integrity number (RIN) > 7.0 was acceptable for cDNA library construction. cDNA libraries were established for each RNA sample using the TruSeq Stranded mRNA Library Prep Kit (Illumina, Inc., San Diego, United States) according to the manufacturer’s instructions. Briefly, the protocol consisted of the following steps. Using oligo (dT) magnetic beads, poly(A)-containing mRNA was purified from 1 µg of total RNA and fragmented into 200–600 bp fragments using divalent cations at 85 °C for 6 min. The cleaved RNA fragments were subsequently subjected to first- and second-strand complementary DNA (cDNA) synthesis. The dUTP mixture was used for second-strand cDNA synthesis, which allowed for the removal of the second strand. The cDNA fragments were end-repaired, A-tailed and ligated with indexed adapters. To remove the second-strand cDNA, the ligated cDNA products were purified and treated with uracil DNA glycosylase. Purified first-strand cDNA was enriched by PCR to create cDNA libraries. Quality control of the libraries was conducted with an Agilent 2200 platform, and the libraries were sequenced with a NovaSeq 6000 platform on a 150 bp paired-end run. By removing the adaptor sequences and low-quality reads, clean reads were obtained. The clean reads were subsequently aligned to the human genome (GRCh38) using HISAT2^17^. For quality control, the raw sequencing data, including the quality distribution of nucleotides, GC content, proportion of PCR duplication, position-specific sequencing quality, and kmer frequency, were evaluated via FAST-QC. HTseq was applied to obtain gene counts, and the RPKM method was used to determine gene expression^18^. The data were analyzed by the DESeq2 algorithm^19^. The genes that were differentially expressed between healthy and unhealthy tendons were considered to have a *P* value < 0.05. The computed *Z* score was used to plot a heatmap of the differentially expressed genes. R (version 4.2.2; Boston, United States) software was used for the data analysis.

### Hallmark gene set analysis and Gene Ontology (GO) analysis of the RNA sequencing data

The top 100 upregulated differentially expressed genes (DEGs) (ranked by fold change) were subjected to enrichment analysis. To evaluate the associations of the various biological events activated in unhealthy tendons, the h.all.v7.4.symbols.gmt sets from the Molecular Signatures Database^20^ were downloaded for the hallmark gene set analysis. For the GO functional analysis, annotations from the R package “org.Hs.eg.db (version 3.1.0)” were retrieved. The R package “clusterProfiler (version 3.14.3)” was used for the enrichment analysis. A *P* value < 0.05 was considered to indicate statistical significance.

### Bioinformatic analysis of the microarray datasets

Microarray datasets were obtained from the Gene Expression Omnibus (GEO) database (ncbi.nlm.nih.gov/geo/) under the ID GSE26051. The data were analyzed using the limma algorithm^21^. Differentially expressed genes between healthy and unhealthy tendons were identified with a *P* value threshold of <0.05. The top 500 upregulated DEGs, ranked by fold change, were subjected to enrichment analysis. GO enrichment analysis was carried out in the same manner as that for the RNA sequencing data. We used GSEA software (version 3.0), which was obtained from the GSEA website^22^ (software.broadinstitute.org/gsea/index.jsp). We downloaded the h.all.v7.4.symbols.gmt subset for hallmark analysis and the c5.go.bp.v7.4.symbols.gmt subset for BP analysis from the Molecular Signatures Database^20^ (http://www.gsea-msigdb.org/gsea/downloads.jsp). The minimum set size was set at 5, and the maximum was set at 5000, with the analysis involving 1000 permutations. A *P* value < 0.05 was considered to indicate statistical significance.

### Portraying the macrophage landscape with xCell

To explore macrophage infiltration in tendon tissues via transcriptomic analysis, the xCell webtool (https://xcell.ucsf.edu/) was used to digitally portray the cellular landscape^23^. Standard RNA-Seq expression was normalized to gene length as transcripts per kilobase million (TPM) and submitted for analysis with the “RNA-seq = True” option. A *P* value of <0.2 was considered to indicate statistical significance according to the instructions of xCell^23^.

### Isolation of mouse tendon cells

The use of the mice was approved, and the mice were housed and maintained in accordance with the Institutional Animal Care and Use Committee guidelines of the University of Western Australia Animal Ethics Committee (RA/3/100/1526; RA/3/100/1731). Six- to eight-week-old C57BL/6 mice were euthanized by cervical dislocation. The middle portions of the Achilles tendons and patellar tendons were isolated and rinsed with phosphate-buffered saline (PBS) containing 100 U/mL penicillin and 100 μg/mL streptomycin (Gibco™, Waltham, United States). Isolated tendon tissue was digested with 3 mg/mL type II collagenase in α-Minimal Essential Medium (MEM Alpha, Gibco™) for 3 h. After filtering through a 70 μm cell strainer, the cells were centrifuged and cultured in Alpha MEM supplemented with 10% fetal bovine serum (FBS, Gibco™), 100 U/mL penicillin, and 100 μg/mL streptomycin. The incubation conditions were 37 °C in a humidified atmosphere containing 5% CO_2_. Passage 3 cells were used in the experiments.

### 3D tendon construct formation and mechanical stimulation

3D tendon construct formation and mechanical stimulation were adapted from published methods^24,25^. Mouse tendon cells were seeded into a T-75 flask (10^6^ cells) and cultured to complete confluence. The cells were then stimulated with 25 ng/mL connective tissue growth factor (CTGF; PeproTech, Waltham, United States) and 4.4 μg/mL ascorbic acid (Sigma‒Aldrich, Burlington, United States) for 6 days to generate an extracellular matrix and to form monolayer cell sheets. Monolayer cell sheets were then detached from the flask and subsequently attached to custom-made tissue hooks in a bioreactor to form 3D tendon constructs^26^. These 3D tendon constructs were cultured in a bioreactor containing Alpha MEM supplemented with 10% FBS, 100 U/mL penicillin, and 100 μg/mL streptomycin. Extracellular vesicles were depleted from the FBS beforehand^27^. For uniaxial 3D stretching, the programmable bioreactor was set up for 8 h/day, followed by 16 h of rest, with 0%, 3%, 6%, or 9% strain, at 37 °C and 5% CO_2_ for 6 days. The strain (in percentage) was calculated as follows: Strain (%) = ((final length – initial length)/initial length) × 100. For example, a 0% strain indicates static culture, whereas a 100% strain implies stretching the tissue to double its original length. The middle thirds of the 3D tendon constructs were obtained for downstream analysis after mechanical stimulation.

### Chemical treatments, transfection, and staining of tendon constructs

To protect the intracellular mitochondrial network in the overloaded tendon constructs, they were incubated with 2.5 mM NAC (Sigma, Burlington, United States) during mechanical stimulation by 9% or 0% strain. To label mitochondria with an RFP in the 3D tendon construct, cell sheets were incubated with Mitochondria-RFP (C10601; CellLight, Thermo Fisher Scientific, Waltham, United States), a baculovirus-packaged DNA construct, for 16 h following the manufacturer’s protocol and then assembled into the bioreactor to form tendon constructs.

To label the mitochondria with MTR and cytoplasm with CFSE for particle characterization, 3D tendon constructs were stained with 1 μM CFSE for 15 min and 20 nM MTR for 30 min before mechanical stimulation. For live-cell imaging to observe mitochondrial morphology in tendon constructs, 3D tendon constructs were stained with 20 nM MTR for 30 min after mechanical stimulation, washed three times, and observed in live-cell imaging solution (A14291DJ, Invitrogen, Waltham, United States).

### Reverse transcription quantitative polymerase chain reaction (RT–qPCR)

Tendon constructs were pulverized into powder using a mortar and pestle under liquid nitrogen conditions. Total RNA was extracted using TRIzol reagent (15596-026, Invitrogen) and a PureLink™ RNA Mini Kit (Invitrogen) according to the manufacturer’s instructions. The RNA concentration was evaluated spectrophotometrically by a NanoDrop 2000 spectrophotometer (Thermo Fisher Scientific). Complementary DNA (cDNA) was synthesized from RNA using Moloney murine leukemia virus (M-MLV) reverse transcriptase (Promega, Madison, United States). The cDNA was subsequently amplified and quantified via real-time PCR using the iQ^TM^ SYBR^®^ Green Supermix (Bio-Rad Laboratories, Hercules, CA, USA). RT‒PCR was repeated using three individual samples from each group, and each sample was analyzed in triplicate. The cycle threshold (Ct) was calculated for each qPCR reaction. The relative expression levels of the target genes were calculated using the comparative 2^-ΔΔCt^ method. The sequences of primers used for the genes are listed in Supplementary Table 2.

### Immunoblot analysis

The tendon constructs were frozen using liquid nitrogen and ground into powder using a mortar and pestle before protein extraction. Tendon constructs or mEP pellets were incubated with radioimmunoprecipitation assay lysis buffer supplemented with protease inhibitor (Roche, Basel, Switzerland) and a phosphatase inhibitor cocktail (Sigma‒Aldrich) for 30 min at 4 °C. After centrifugation, the debris was pelleted, and the supernatants were transferred into 1 mL tubes, which were subsequently diluted with 4 × SDS sampling buffer and boiled for 5 min. The protein concentration was evaluated via a Bio-Rad protein assay (Bio-Rad, Hercules, United States). Unless otherwise stated, equal amounts of protein were separated via SDS‒polyacrylamide gel electrophoresis and subsequently transferred to nitrocellulose membranes (Millipore). The membranes were incubated at 4 °C overnight with primary antibodies against β-actin JLA20 (1:5000; Developmental Studies Hybridoma Bank, Iowa City, United States), VDAC (1:1000; ab15895, Abcam, Cambridge, United Kingdom), TOM20 (1:1000; ab186735, Abcam), CD63 (1:1000; ab217345, Abcam), Annexin A2 (1:1000; #8235, Cell Signaling, Danvers, United States), Lamin A/C (1:2000; #4777, Cell Signaling), and Albumin (1:1000; ab175934, Abcam) and then incubated with the corresponding secondary antibodies conjugated with horseradish peroxidase (Sigma‒Aldrich) for 1 h at room temperature. Immunoreactivity was evaluated by the Western Lightning Ultra Detection Kit (PerkinElmer, Waltham, Massachusetts, United States) according to the manufacturer’s instructions. Detection of chemiluminescence was performed with a ChemiDoc™ MP Imaging System (Bio-Rad). Uncropped images are provided in Supplementary Fig. 9. Quantification was performed with ImageJ (National Institutes of Health, Bethesda, United States).

### Finite element analysis (FEA)

To illustrate the uneven strain distribution in the tendon construct, a two-dimensional plane stress finite element model of the sample was generated via simulation via the ANSYS software package (ANSYS, Canonsburg, USA; v2021R1). The construct was modeled in a concave shape to match the geometry of the experimental sample, which was assumed to be isotropic, linearly elastic, and incompressible in the simulation. A Young’s modulus of 10 GPa (a typical value obtained from the stress‒strain curve of tendon^28^) was used as input in the simulation. The 2-D model was generated by dividing the geometry into mesh elements with a mesh size of 0.2 mm, in which the approximate solutions to the partial differential equations describing the mechanical response of the construct subjected to a known applied strain of 3% were computed, with a well-defined (fixed) boundary condition assigned to one end of the construct.

### Tendon-derived mEP collection

mEPs were isolated from the conditioned media of tendon constructs according to published methods^29^. Briefly, tendon constructs were cultured in a bioreactor containing 30 mL of Alpha MEM for each tendon construct supplemented with 10% FBS, 100 U/mL penicillin, and 100 μg/mL streptomycin. Extracellular vesicles in the FBS were depleted beforehand^27^. After mechanical stimulation for 6 days, conditioned media were obtained and centrifuged at 400 × g to pellet nonadherent cells for 5 min. The supernatant was subsequently centrifuged to pellet cellular debris at 1500 × g for 10 min. The remaining supernatant was subsequently centrifuged to pellet mEPs at 18000 × g for 30 min. To separate the mEP fractions through a high-resolution iodixanol density step-gradient, the pellet containing mEPs was resuspended in a 40% (v/v) iodixanol (OptiPrep, Burlington, United States) solution. A density step gradient was set up by carefully layering decreasing concentrations of iodixanol (20, 15, 13, 11, 9, 7, and 5%) on top of the 40% mEPs containing iodixanol solution. The column was then centrifuged for 16 hs at 4 °C at 200,000 × g. Afterward, 1.25 mL of the gradient from the top (fraction 1) was collected, and 1.5 mL of the fractions (fractions 2–8) were subsequently collected. All the fractions were mixed with 4 mL of ice-cold PBS, except for Fraction 8, which was mixed with 8 mL of ice-cold PBS. The fractions were subsequently centrifuged at 18000 × g for 30 min to collect the pellets of each fraction.

### Chemotaxis assay and cytokine production analysis

The RAW 264.7 mouse macrophage line was used for the subsequent experiments. The chemotaxis of RAW 264.7 cells was assessed with a colorimetric cell migration assay kit (ECM508, MILLIPORE QCM^TM^, Sigma) based on the Boyden chamber principle, with inserts utilizing polycarbonate membranes with 8 μm pores. Briefly, RAW 264.7 cells were starved by incubating them for 24 h prior to the assay in serum-free Dulbecco’s modified Eagle’s medium (DMEM, ATC302002, ATCC, Manassas, United States). Then, 300 μL of 1 × 10^6^ RAW 264.7 cells was added to the insert. Then, 500 μl of serum-free DMEM was added to the lower chamber, with either PBS as a negative control or mEPs in PBS as a chemoattractant. To evaluate the biological function of the mEPs, mEPs from 500 μm tendon constructs in 500 μL medium were applied to each insert; for a tendon construct with a length of 1 cm, one-twentieth of the mEPs were used. The mass of the 500 μm tendon construct was found to be 1.1 ± 0.1 mg, with the protein amount shown as mEP_0%_ (5.85 ± 0.18 µg), mEP_3%_ (5.87 ± 0.34 µg), mEP_6%_ (6.94 ± 0.21 µg), and mEP_9%_ (8.88 ± 0.30 µg). After incubating for 24 h at 37 °C in 5% CO_2_, the remaining cell suspension from the top chamber of the insert was removed. The migration insert was placed into a well containing the cell stain from the kit and left for 20 min at room temperature. The nonmigratory cell layer from the interior of the moist insert was then removed with cotton-tipped swabs. The inserts were then air-dried and observed by an Eclipse TE2000-S microscope (Nikon, Konan, Japan). For quantification, the stained insert was moved to a well containing extraction buffer for 15 min at room temperature, after which the mixture was gently tilted back and forth during incubation to extract the stain from the underside. The optical density (OD) of the extracted stain was measured at 560 nm by a Cell Imaging MultiMode Reader (Cytation 5, BioTek, Winooski, U.S.) with Gen5 software (BioTek).

To evaluate the ability of tendon-derived mEPs to induce the production of inflammatory cytokines, RAW 264.7 cells were cultured with tendon-derived mEPs, PBS (as the negative control), or 500 ng/mL lipopolysaccharide (LPS) (as the positive control) for 8 h in 6-well plates. The concentration of mEPs was the same as that used in the chemotaxis assay. However, to examine whether mEPs from various conditions mediate the release of inflammatory cytokines in a dose-dependent manner, we used a 10-fold mEP concentration gradient. These included mEPs from tendon constructs of 50 μm, 500 μm, and 5 mm in 500 μL medium. FBS-free media supplemented with 200 μg/mL bovine serum albumin (BSA) were used for culture. Conditioned media were then collected for cytokine measurements using a bead-based multiplex assay panel, LEGENDplex^TM^ Mouse Macrophage Panel (13-plex) (740845; BioLegend, San Diego, United States), per the manufacturer’s instructions. Briefly, the samples were incubated with the capture beads for 2 h at room temperature on a plate shaker. Then, the beads were spun down and washed. Detection antibodies were added to the beads, which were incubated for 1 h at room temperature, followed by incubation with SA-PE at room temperature on a plate shaker. The beads were then spun down, washed and resuspended in wash buffer. As the beads are differentiated by size and internal fluorescence intensity, analyte-specific populations can be identified and quantified by flow cytometry (FACS Canto II Flow Cytometry, BD, Franklin Lakes, United States). The instrument was set up according to the manufacturer’s protocol. The concentration of each cytokine was analyzed by a standard curve generated in the same assay using the standard samples provided in the assay panel. The data were analyzed with LEGENDplex™ software (BioLegend).

### mEP characterization by flow cytometry and dynamic light scattering

For mEP characterization, nanoparticles with 4 different diameters (220 nm, 450 nm, 880 nm, and 1340 nm) were applied for the calibration of size with an LSR Fortessa flow cytometer (BD) using SSC measurements (voltage: 412; gain: 1.0). For lipophilic membrane identification of mEPs, mEPs were stained with PKH26 (MINI26, Sigma‒Aldrich) after collection following the manufacturer’s protocol. Briefly, mEPs were suspended in Diluent C (CGLDIL, Sigma). Then, the mEPs were stained with 2 × 10^–6^ M PKH26 for 5 min. mEPs containing only the diluent were used as the control. The mEPs were then analyzed by an LSR Fortessa flow cytometer (BD) (voltage: 342; gain: 1.0). The data were processed and analyzed with FlowJo software (V10, BD Life Sciences, Franklin Lakes, United States).

Dynamic light scattering is a commonly used method for determining the size of small particles. To characterize the size of the mEPs by dynamic light scattering, the mEPs were suspended in cuvettes and measured by a Zetasizer Nano Series (Malvern, United Kingdom) with Zetasizer Software (version 7.13, Malvern). The refractive index value “n” was set as 1.40, and the absorption value “k” was set as 0.01.

### Depletion of the mitochondria from tendon-derived mEPs

Free mitochondria were depleted from mEPs by MACS. Tendon-derived mEPs were incubated with anti-rabbit IgG magnetic beads coupled with anti-TOM20 antibodies (Miltenyi Biotec, Bergisch Gladbach, Germany). Then, suspensions of magnetically labeled mEPs or sham-labeled mEPs were applied to an LD column (130-042-901; Miltenyi Biotec) in the magnetic field of a MidiMACS Separator (130-042-302; Miltenyi Biotec). The flow through the LD column included TOM20- mEPs. The magnetically labeled TOM20+ mEPs were retained in the column and then eluted after the column was removed from the separator.

### Confocal laser scanning microscopy (CLSM) imaging and processing

For live-cell imaging of mitochondrial morphology, pieces of the middle portion of the tendon constructs were cut out and transferred from the bioreactor to 35-mm glass-bottom Petri dishes (P35G-0.17-14-C; MatTek, Ashland, United States). The mEP imaging procedure was modified from a published method^30^. The mEPs in SlowFade^TM^ Glass Antifade Mountant (S36917, Thermo Fisher Scientific) were applied to 35-mm glass-bottom Petri dishes (P35G-0.17-14-C, MatTek) and flattened against the surface tension before imaging.

To observe the morphology of RAW 264.7 cells after treatment with or without ExtraMito-containing mEPs, 3D tendon constructs were stained with 20 nM MTR for 30 min before mechanical stimulation. Then, MTR-labeled mEP_9%_ was obtained from MTR-labeled tendon constructs after 6 days of mechanical stimulation with 9% strain. RAW 264.7 cells were prelabeled with 1 μM CFSE for 15 min. CFSE-labeled RAW 264.7 cells were then cultured with or without MTR-labeled mEP_9%_ on 13 mm microscope coverslips (ProSciTech, Kirwan, Australia) placed in 24-well plates. The concentration of mEPs was the same as that used in the chemotaxis and cytokine production assays. After 24 h of incubation, the RAW 264.7 cells were washed with PBS and fixed in 4% paraformaldehyde (PFA). Hoechst 33342 (1:5000; 62249; Thermo Fisher Scientific) was then applied for nuclear staining. The coverslips were mounted with ProLong Diamond Antifade Medium (P36970, Invitrogen).

Images were acquired on a Nikon A1Si confocal microscope with a 60× water immersion objective or a Nikon A1R confocal microscope with a 100x oil immersion objective. The CLSM instrument was equipped with a perfect focus system (PFS) and a Tokai Hit incubation chamber for live-cell imaging, set at 37 °C and 5% CO_2_. Three-dimensional images were acquired by *Z*-stacks with 0.1–0.15 μm interstack intervals. Denoising, deconvolution, 3D rendering, and quantification of images were performed using NIS-Elements Advanced Research (Nikon), Imaris (Bitplane, Zurich, Switzerland), and ImageJ (National Institutes of Health, Bethesda, United States).

### Transmission electron microscopy (TEM)

The mEPs were fixed in a mixture of 2.5% glutaraldehyde and 2% paraformaldehyde in 0.1 M sodium cacodylate buffer (pH 7.4) (CEMS15960-01, ProSciTech). Carbon-coated copper 200 mesh grids (GSCU200CC, ProSciTech) were glow discharged in a PELCO easiGlow (Ted Pella, Inc., Redding, United States). Fixed mEPs were then added to the glow-discharged grids held by self-clamping tweezers for 1 min. The solution was then removed with Whatman filter paper, and the grids were negatively stained with 1% (w/v) uranyl acetate (Electron Microscopy Sciences, Hatfield, Pennsylvania) for 30 s. An F200 transmission electron microscope (JEOL, Tokyo, Japan) with a Camera OneView 4k (Gatan, Pleasanton, United States) and DigitalMicrograph (Gatan) was used for imaging at 200 kV.

### Statistical analysis

GraphPad Prism software (version 8, GraphPad Software, Inc., San Diego, United States) was used for statistical calculations. A two-tailed Student’s *t* test was performed for means comparisons between two groups. For comparisons of multiple group data, one-way analysis of variance (ANOVA) with Tukey’s post hoc test was used. At least three biological replicates were performed for each experiment. In each imaging experiment, at least five different areas were scanned for each biological replicate. The data are presented as the means ± SEMs. A *P* value < 0.05 was considered to indicate statistical significance and is marked by asterisks (ns, no significance; *P < 0.05; **P < 0.01; and ***P < 0.001).

## Results

### Transcriptomic profiling reveals oxygen-related reactions, extracellular particles, and inflammation in human tendinopathic tissues

**Fig. 1:**
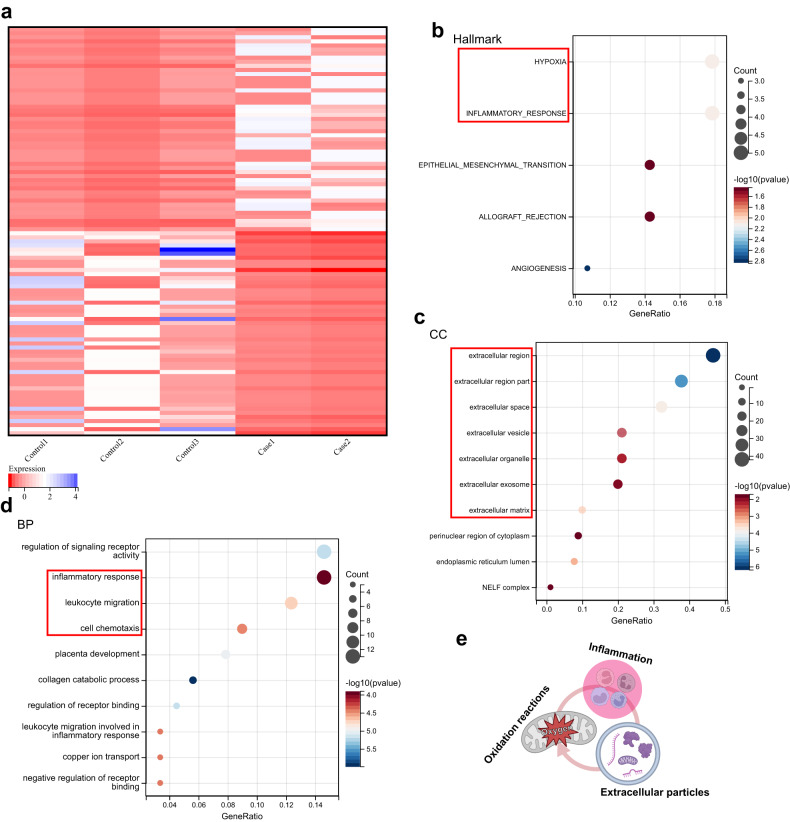
RNA sequencing reveals that oxygen-related reactions, extracellular particles, and inflammation are present in human tendinopathy. **a** Heatmap of the top 50 upregulated genes and the top 50 downregulated genes in human tendons with tendinopathy (*n* = 2) compared to healthy human tendons (*n* = 3). The color indicates the *z* score of the genes’ transcripts per million. **b**, **d** Hallmark gene set analysis (**b**), Gene Ontology (GO) cellular component analysis (CC) (**c**), and Gene Ontology (GO) biological process (BP) analysis (**d**) of the top 100 upregulated genes in tendons from patients with tendinopathy compared to healthy tendons. The top-ranked terms according to *P* values (all <0.05) are displayed in bubble plots. **e** Hypothetical model of the underlying mechanism of tendinopathy. Oxygen-related reactions, inflammation and extracellular particles collectively induce tendinopathy. N, biological replicates.

To investigate the subcellular responses in tendinopathy, we performed RNA-seq to obtain transcriptomic profiles from healthy human tendons and tendinopathic tissues. Healthy tendon tissues were obtained from 3 male donors. Diseased tendons were obtained from a male patient and a female patient with chronic tendinopathy. Transcriptomic profiling identified 1083 upregulated genes and 1261 downregulated genes (*P* value < 0.05, |logFoldChange | > 2). The expression of the top 50 upregulated genes and the top 50 downregulated genes is shown in a heatmap (Fig. 1a, Supplementary Table 1). To evaluate the biological events activated in tendons with tendinopathy, we performed enrichment analysis of the hallmark gene sets of the top 100 upregulated genes in tendons with tendinopathy. The results revealed that “hypoxia” and “inflammatory response” were the top-ranked biological events (Fig. 1b). For functional analysis, we used the Gene Ontology (GO) cellular component (CC) and biological process (BP) terms. GO CC analysis of the top 100 upregulated genes revealed that the DEGs were enriched in extracellular components (Fig. 1c). We also noted that the highest ranked cellular parts were “extracellular vesicle” and “extracellular organelle” according to the GO CC analysis. The GO BP analysis of these genes indicated that the highly ranked functional activities were related to the inflammatory response, including “leukocyte migration” and “cell chemotaxis” (Fig. 1d).

We then accessed archived microarray data (GSE26051) from the Gene Expression Omnibus (GEO) database. These microarray data included 23 diseased tendons with tendinopathy and 23 normal tendons as controls. The enrichment analysis of the microarray data supported our RNA sequencing findings. Gene Set Enrichment Analysis (GSEA) of the microarray data revealed that several inflammatory responses, such as “positive regulation of leukocyte adhesion of vascular endothelial cell” and “positive regulation of leukocyte tethering or rolling”, were significantly activated in tendinopathy (Supplementary Fig. 1a). Furthermore, GSEA of hallmark gene sets showed that enrichment in “hypoxia” was positively associated with tendinopathy (Supplementary Fig. 1b). GO CC analysis of the top 500 genes upregulated in the tendons from patients with tendinopathy compared to healthy individuals revealed that most of the genes activated in patients with tendinopathy were significantly related to extracellular regions and extracellular particles, including different extracellular components (Supplementary Fig. 1c). Taken together, these data indicated that oxygen-related reactions, extracellular particles, and the inflammatory response may play essential roles in the pathology of tendinopathy (Fig. 1e).

### A bioreactor platform mimics the mechanical environment of the tendon

**Fig. 2:**
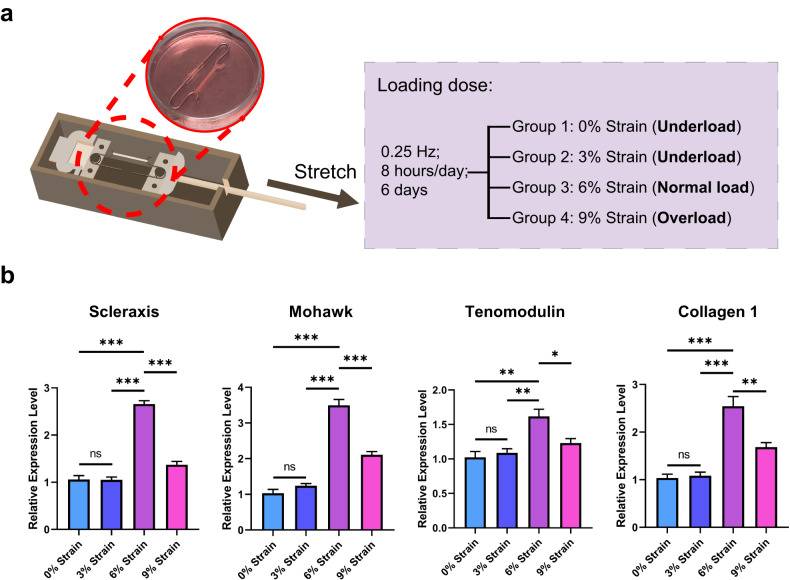
The mouse tendon construct simulates tendon loading in a 3D mechanical environment. **a** Schematic diagram of the mechanical stimulation of 3D mouse tendon constructs at different loading degrees. **b** The relative expression levels of the tenogenic-related genes Scleraxis, Mohawk, Tenomodulin, and Collagen 1 were measured via RT‒qPCR. 36B4 expression was measured as an internal control. The results are presented as three biological replicates from three independent experiments. The data are presented as the means ± SEMs. One-way ANOVA with Tukey’s multiple comparisons test was used for statistical analysis. ***P < 0.001; **P < 0.01; *P < 0.05; ns, not significant.

To further explore the relationships among oxygen-related reactions, inflammation, and extracellular particles, we established an in vitro model to closely mimic the in vivo mechanical environment and to clearly observe tendon cell function in tendinopathy. This model enabled us to apply 3D force to tendon constructs in a bioreactor (Fig. 2a)^25,31^. We applied four cyclic stretching regimens of 0% (static culture), 3%, 6%, or 9% strain (0.25 Hz; 8 h/day; 6 days) to the tendon constructs. Consistent with our previous study on an ex vivo rabbit tendon model^26^, examination of tenogenic-related genes for evaluating cell function, including Scleraxis (*Scx*), Mohawk (*Mkx*), Tenomodulin (*Tnmd*), and Collagen 1 (*Col1a1*), by reverse transcription–quantitative polymerase chain reaction (RT–qPCR) showed that, compared with static culture with 0% strain, 6% strain increased the expression level of *Scx* (2.51-fold change, FC), *Mkx* (3.39 FC), *Tnmd* (1.58 FC), and *Col1a1* (2.45 FC) in tendon constructs (Fig. 2b), indicating that optimal loading with 6% strain was beneficial for cell function. However, compared with the 6% strain, the 9% strain decreased the expression levels of *Scx* (0.52 FC), *Mkx* (0.60 FC), *Tnmd* (0.76 FC), and *Col1a1* (0.66 FC) in the tendon constructs, revealing that 9% strain was overload that impaired cell function. The expression levels of *Scx*, *Mkx*, *Tnmd*, and *Col1a1* did not significantly change in the tendon constructs receiving 3% strain compared to those in the tendon constructs receiving 0% strain. Together with the findings of previous work^26^, we conclude that 0% strain and 3% strain underload mouse tendon constructs, 6% strain is a normal loading percentage, and that 9% strain overloads and simulates the mechanical overload environment in tendinopathy.

### 3D cyclic strain dose-dependently affects the mitochondrial network in tendon constructs

**Fig. 3:**
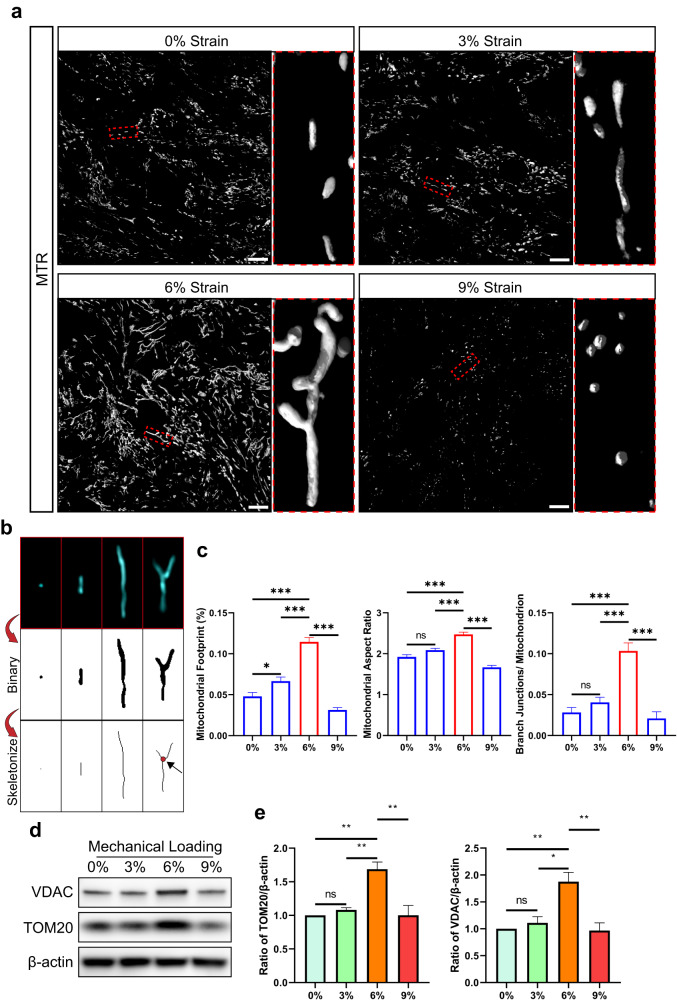
Three-dimensional cyclic strain affects the mitochondrial network in tendon cells. **a** Representative 3D live-cell confocal images of MitoTracker Red (MTR)-labeled mitochondria in 3D tendon constructs after 0%, 3%, 6% or 9% cyclic strain. Scale bar, 10 μm. **b** Method for the quantitative analysis of mitochondrial morphology. Four common mitochondrial morphologies, namely, puncta, rods, elongated mitochondria and mitochondrial interconnected networks, were observed in the tendon constructs. After binaryization and skeletonization, branch junctions (the red dot indicated by the black arrow) and branches could be identified in the mitochondrial interconnected network. **c** Characterization of mitochondrial morphology after 0%, 3%, 6% or 9% cyclic strain by calculating the percentage of the mitochondrial footprint (left), the mitochondrial aspect ratio (middle), and the number of branch junctions per mitochondrion (right) in 3D tendon constructs (*n* = 15 each group). **d** Immunoblot analysis of VDAC and TOM20 protein levels in 3D tendon constructs subjected to 0%, 3%, 6% or 9% cyclic strain. **e** Quantitative analysis of three biological replicates from three independent experiments. β-Actin expression was measured as the internal control. The data are presented as the means ± SEMs. One-way ANOVA with Tukey’s multiple comparisons test was used for statistical analysis. ***P < 0.001; **P < 0.01; *P < 0.05; ns not significant.

We then investigated the impact of underload, normal load, and overload on oxygen-related reactions in the tendon constructs. Mitochondria, as the powerhouses of cells, control the response to oxygen^32^. To study the effects of cyclic strains on oxygen-related reactions, we used live-cell CLSM to observe the morphology of MitoTracker Red (MTR)-labeled mitochondria in 3D mouse tendon constructs (Fig. 3a). Volume-rendered 3D reconstruction of confocal images showed that in an underloaded environment with 0% strain or 3% strain, the mitochondria tended to be rod-like and puncta-like. A normal load of 6% strain could induce the formation of elongated mitochondria and activate the mitochondrial network. The mitochondrial network has been reported to increase energy production, maintain mitochondrial homeostasis, and serve as a signal of active mitochondrial dynamics^33–35^. In tendon constructs overloaded with 9% strain, the mitochondria were fragmented and appeared to be rounded and punctate. Quantitative analysis revealed the largest mitochondrial footprint, highest mitochondrial aspect ratio, and greatest number of branch junctions per mitochondrion in the tendon constructs receiving 6% strain, compared to those receiving 0%, 3%, and 9% strain, which indicated the highest mitochondrial content, longest mitochondria, and most mitochondrial networks, respectively (Fig. 3b, c). However, although there was no significant difference in the tenogenesis of the tendon constructs of the 0% strain and 3% strain groups (Fig. 2b), we found that 3% strain still increased the mitochondrial footprint in the tendon constructs compared to that in the 0% strain group according to quantitative analysis of mitochondrial morphology (Fig. 3c). Further assays for mitochondrial homeostasis were conducted by immunoblotting for two mitochondrial markers, voltage-dependent anion channel (VDAC) and translocase of outer mitochondrial membrane 20 (TOM20) (Fig. 3d, e). Consistent with the findings for the tenogenic-related genes, the immunoblot results revealed the highest expression of VDAC and TOM20 in the 6% strain group, confirming that the greatest amount of mitochondria was present in the tendon constructs receiving a normal load. Increased mitochondrial content in tendon constructs receiving 3% strain compared to 0% strain was not reproducibly detected by immunoblotting. This discrepancy might be because the size of the tendon constructs we used for immunoblotting was greater than the size we scanned in the microscopy experiments, resulting in less mean strain in the sample section, which was below a certain threshold required for mitochondrial reaction (Supplementary Fig. 2). Taken together, these results show that cyclic strain has an impact on mitochondria in tendon cells. Compared to underloading, normal loading increases the mitochondrial content and activates the mitochondrial network in tendon cells, while overloading eliminates this effect.

### Cyclic strain induces the release of extracellular mitochondrial (ExtraMito) particles from tendon cells

**Fig. 4:**
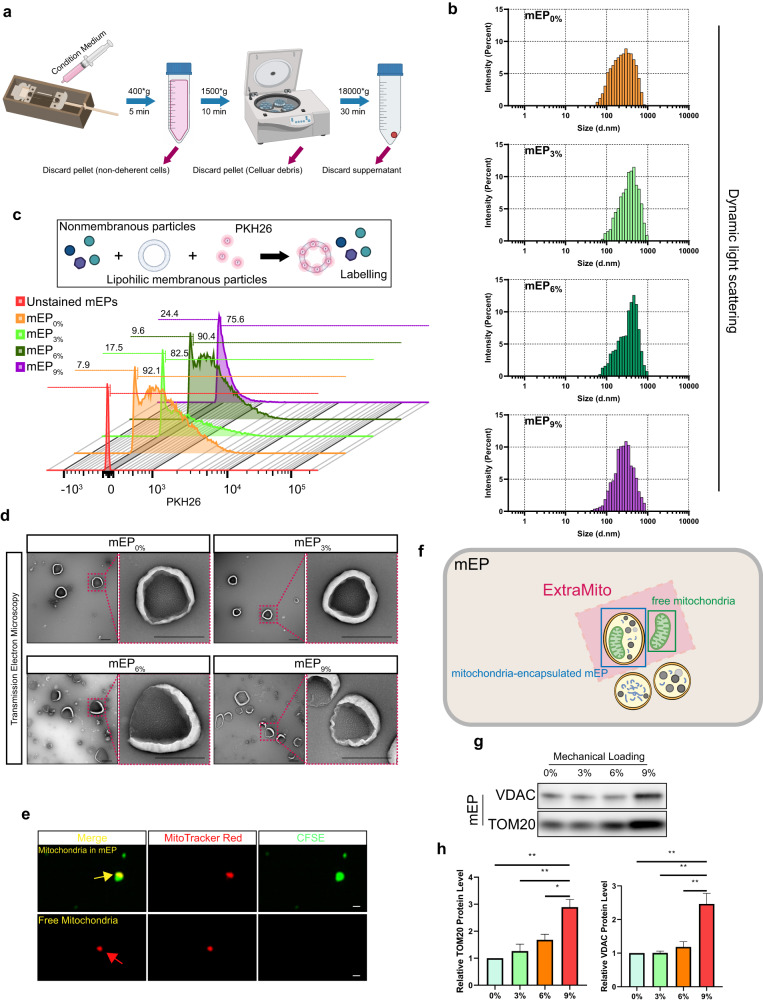
The cyclic strain induces the release of ExtraMito particles from tendon cells. **a** Schematic diagram of the collection of medium extracellular particles (mEPs) by differential centrifugation. **b** Histogram of the size distribution of mEPs from tendon constructs receiving 0%, 3%, 6%, or 9% cyclic strain (mEP_0%_, mEP_3%_, mEP_6%_, or mEP_9%_), characterized by dynamic light scattering. **c** Histogram of mEPs from tendon constructs receiving 0%, 3%, 6%, or 9% cyclic strain poststained with PKH26 after collection, characterized by flow cytometry. **d** Representative transmission electron microscopy images of mEPs. Scale bar, 500 nm. **e** Confocal fluorescence microscopy image showing two forms of ExtraMito particles, namely, mitochondria (red, prestained with MitoTracker Red), encapsulated in mEPs (green, prestained with CFSE) (upper panel), and free extracellular mitochondria (bottom panel). Scale bar, 1 μm. **f** A schematic diagram explaining the terminology used for mEP, ExtraMito particles, free mitochondria and mitochondria-encapsulated mEP and their relationships in the present study. **g** Immunoblot analysis of VDAC and TOM20 protein levels in mEPs from tendon constructs subjected to 0%, 3%, 6%, or 9% cyclic strain and (**h**) quantitative analysis of three biological replicates from three independent experiments. The data are presented as the means ± SEMs. One-way ANOVA with Tukey’s multiple comparisons test was used for statistical analysis. **P < 0.01; *P < 0.05.

Previous studies have reported that ExtraMito particles are intercellular communicators that can affect inflammatory responses^29,36^. As the RNA sequencing data indicated the potential relationships between extracellular particles, inflammation and tendinopathy, we hypothesized that mitochondria affected by different levels of cyclic strains could be further released into the extracellular environment as particles and affect the immune microenvironment. To date, little is known about the capacity of tendon cells to release mitochondria into the extracellular environment. We then explored the existence and regulation of tendon-derived ExtraMito particles. As ExtraMito particles are a subtype of mEP^29^, we first collected mEPs from the conditioned media of tendon constructs stimulated by different levels of cyclic strain, including 0% strain, 3% strain, 6% strain and 9% strain, in the bioreactors. The mEPs were obtained by differential centrifugation (Fig. 4a). Flow cytometry showed that the sizes of these mEPs were similar among the different degrees of strain and were mainly between 220 nm and 880 nm (Supplementary Fig. 3). The size of the particles was further characterized by dynamic light scattering, a technique commonly used for nanoparticle characterization. The results confirmed that the particles obtained were mainly between 100 nm and 1000 nm in size (Fig. 4b). Poststaining of mEPs revealed that most of the mEPs could be stained with PKH26, a lipophilic membrane dye, revealing the presence of a membrane in most of these mEPs (more than 75%) (Fig. 4c). The mEPs were also characterized by transmission electron microscopy (Fig. 4d). We further assessed proteins from different cellular compartments and found that CD63 (cell membrane marker), Annexin A2 and β-actin (cytoplasmic marker) were in high concentrations in the tendon-derived mEPs, while LaminA/C (nuclear marker) and Albumin (protein from culture medium) were in low abundance (Supplementary Fig. 4). To detect the ExtraMito particles in mEPs, we then prestained mitochondria with MitoTracker Red (MTR) and cytoplasm with carboxyfluorescein succinimidyl ester (CFSE) in tendon constructs before applying mechanical stimulation. After 6 days of cyclic stretching, CLSM revealed that ExtraMito particles were released by the tendon constructs, regardless of the degree of strain (Fig. 4e). There were two forms of ExtraMito particles, namely, mitochondria-encapsulated mEPs and free extracellular mitochondria (Fig. 4f). To exclude the possibility of MTR leakage, we transfected tendon constructs with red fluorescent protein (RFP) fused to mitochondria instead of MTR. After 6 days of cyclic stretching in the bioreactor system, fluorescence confocal imaging confirmed the presence of the two forms of ExtraMito particles from the conditioned media (Supplementary Fig. 5). To quantify the amount of ExtraMito particles released from tendon constructs in different mechanical environments, we examined the mitochondrial markers VDAC and TOM20 at the protein level (Fig. 4g, h). Immunoblotting revealed that the highest level of ExtraMito particles was produced by tendon constructs that received 9% strain (ExtraMito_9%_). Collectively, these data suggest that tendon cells can release ExtraMito particles, which is enhanced by mechanical overload.

### Extracellular particles from tendon cells induce macrophage infiltration and cytokine release

**Fig. 5:**
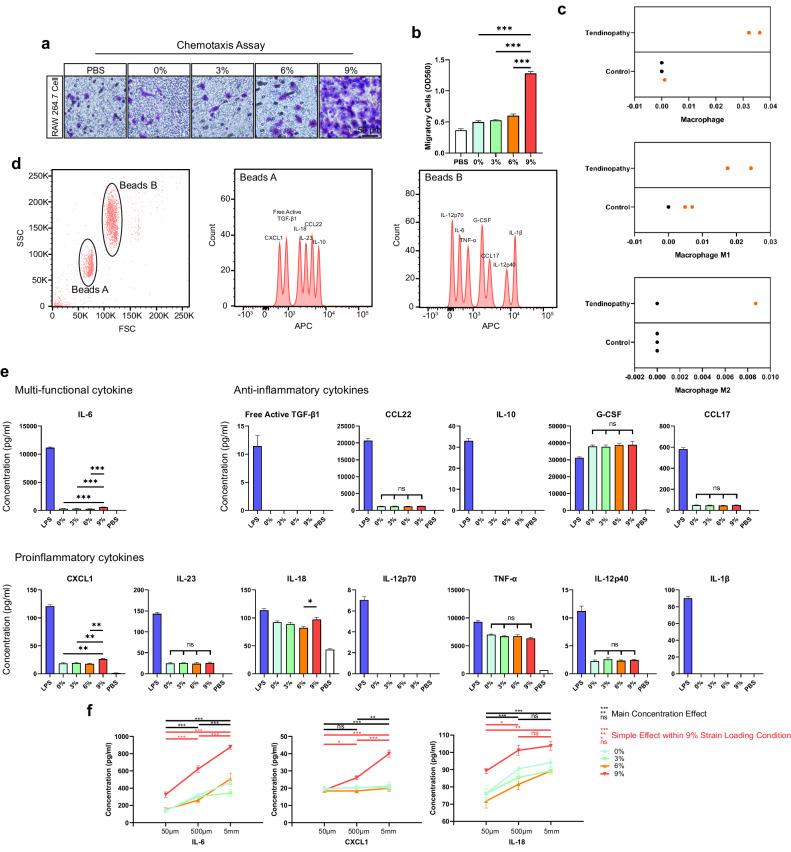
Tendon-derived mEPs can induce macrophage infiltration and cytokine release. **a** Chemotaxis assay for RAW 264.7 cells toward tendon-derived mEPs and (**b**) corresponding quantitative colorimetric measurements are presented as three biological replicates from three independent experiments. RAW 264.7 cells that migrated to the bottom of the insert microporous membrane in the Boyden chamber were stained. The mEPs from tendon constructs receiving 0%, 3%, 6%, or 9% strain were used as chemoattractants in the lower chamber. Scale bar, 50 μm. **c** xCell analysis of bulk RNA sequencing data from healthy tendons and tendons with tendinopathy showing macrophage infiltration in diseased human tendons. The dots in orange indicate that *P* < 0.2. **d** Gating strategy for identification of different cytokines by a bead-based immunoassay. A total of 13 bead populations were distinguished by size and allophycocyanin (APC) fluorescence. **e** Bead-bas**e**d immunoassay showed that the mEPs from tendon constructs that received 0%, 3%, 6%, or 9% strain induced macrophages to secrete different cytokines (*n* = 3 per group). Lipopolysaccharide (LPS) (500 ng/mL) was used as the positive control to induce cytokine release from RAW 264.7 cells. Phosphate-buffered saline (PBS) as the carrier was used as another control. **f** The bead-based immunoassay showed that mEPs mediated RAW 264.7 cells to release IL-6, CXCL1 and IL-18 in a dose-dependent manner (*n* = 3 each group). Two-way ANOVA with Tukey’s multiple comparisons test was used for statistical analysis of the main concentration effect at different doses. Two-way ANOVA with Tukey’s multiple comparisons test was used to compare the effects of different doses within mEP_9%_ for simple effect analysis. The data are presented as the means ± SEMs. One-way ANOVA with Tukey’s multiple comparisons test was used for statistical analysis. ***P < 0.001; **P < 0.01; *P < 0.05; ns, not significant.

We further explored the relationship between inflammation and mEPs, including ExtraMito particles. Macrophage infiltration and increased proinflammatory cytokine production have been reported as two major pathological features of tendinopathy^37,38^. Our RNA sequencing data also showed that “leukocyte migration” and “cell chemotaxis” were activated in tendinopathy (Fig. 1d). Here, the effects of mEPs, including ExtraMito particles, on macrophage infiltration were first tested. We performed a chemotaxis cell migration assay with the RAW 264.7 macrophage line for mEPs released from 3D tendon cell constructs in different loading environments in a migration chamber system based on the Boyden chamber principle (Fig. 5a, b). RAW 264.7 cells exhibited significantly greater chemotaxis toward mEPs excreted by tendon constructs receiving 9% strain (mEP_9%_) than those excreted by tendon constructs receiving mEP_0%_, mEP_3%_ or mEP_6%_, which was consistent with previous reports showing that overload could induce significant macrophage infiltration and tendinopathy^2,39^. To validate the macrophage infiltration results, we analyzed the transcriptomic profiles of healthy human tendons and tendons with tendinopathy via xCell, a gene signature-based method for inferring the content of infiltrated macrophages in tendon tissues^23^. The results revealed an increase in the number of macrophages in the tendons with tendinopathy, which also indicated that overload induced macrophage infiltration into the tendon microenvironment (Fig. 5c).

To evaluate the ability of mEPs from tendon cells to induce the secretion of inflammatory cytokines from macrophages, 13 key cytokines from the conditioned media of RAW 264.7 cells were evaluated after culturing RAW 264.7 cells with mEP_0%_, mEP_3%_, mEP_6%_, or mEP_9%_. Cytokines including multifunctional cytokine Interleukin 6 (IL-6), proinflammatory cytokines C-X-C Motif Chemokine Ligand 1 (CXCL1), IL-18, IL-23, IL-12p70, Tumor Necrosis Factor α (TNF-α), IL-12p40, and IL-1β, and anti-inflammatory cytokines Free Active Transforming Growth Factor β1 (TGF-β1), C-C Motif Chemokine Ligand 22 (CCL22), IL-10, Granulocyte Colony Stimulating Factor (G-CSF), and C-C Motif Chemokine Ligand 17 (CCL17), were tested by a bead-based multiplex assay panel with fluorescence-encoded beads and flow cytometry (Fig. 5d). Our results showed that RAW 264.7 cells released increasing amounts of IL-6, CCL22, GCSF, CCL17, CXCL1, IL-23, IL-18, TNF-α and IL-12p40 when treated with mEPs from mouse tendon cells, compared to PBS-treated RAW 264.7 cells (Fig. 5e). Among these cytokines, the secretion levels of IL-6, CXCL1 and IL-18 varied when mEPs from tendons subjected to different loading environments were applied. Interestingly, it has been reported that IL-6, CXCL1 and IL-18 can induce inflammatory reactions^40,41^, indicating that the mechanical messages carried by mEPs can be passed to the immune microenvironment and mainly affect proinflammation. Moreover, compared to mEP_0%_, mEP_3%_ and mEP_6%,_ mEP_9%_ induced the highest secretion of IL-6 and CXCL1 from RAW 264.7 cells. As tendon cells have been reported to synthesize cytokines^13^, we further directly examined the cytokines inside mEPs. Our data showed that the levels of IL-6, CXCL1 and IL-18 within mEPs were lower than the detectable levels (Supplementary Fig. 6), indicating that these cytokines were mainly secreted from RAW 264.7 cells rather than from mouse tendon mEPs. To examine whether mEPs from various conditions mediate the release of inflammatory cytokines in a dose-dependent manner, we cultured RAW 264.7 cells with varying doses of mEP_0%_, mEP_3%_, mEP_6%_ and mEP_9%_. We used a 10-fold mEP concentration gradient. The analysis of the conditioned media using the bead-based multiplex assay indicated that, considering the general concentration effect, mEPs mediated the release IL-6, CCL22, G-CSF, CCL17, CXCL1, IL-23, IL-18, TNF-α, and IL-12p40 by RAW 264.7 cells in a dose-dependent manner (Supplementary Fig. 7). The ability of mEP_0%_, mEP_3%_, mEP_6%_ and mEP_9%_ to induce the secretion of most cytokines exhibited similar trends at different doses. Notably, mEP_9%_ significantly induced the highest expression levels of IL-6 and IL-18 compared to those induced by mEP_0%,_ mEP_3%_, and mEP_6%_ at different doses (Fig. 5f). However, at a low dose, mEP_9%_ did not induce higher CXCL1 secretion than mEP_0%_, mEP_3%_, or mEP_6%_. The simple effect analysis of cells treated with different mEPs revealed a significant increase in CXCL1 expression levels when treating with mEP_9%_ at higher doses. This trend was not observed with the other mEP treatments, suggesting that mEP_9%_ has specific effects on inducing CXCL1 expression that cannot be compensated for by increasing the quantity of mEP_0%_, mEP_3%_, or mEP_6%_. These data suggested that mEPs released from tendon cells stimulated by different degrees of strain could induce RAW 264.7 macrophages to secrete proinflammatory cytokines. Taken together, these results suggest that mEPs secreted from overloaded tendon cells induce macrophage chemotaxis and increase the secretion of the proinflammatory cytokines IL-6 and CXCL1 from macrophages.

### Tendon-derived ExtraMito particles are essential for the induction of macrophage chemotaxis

**Fig. 6:**
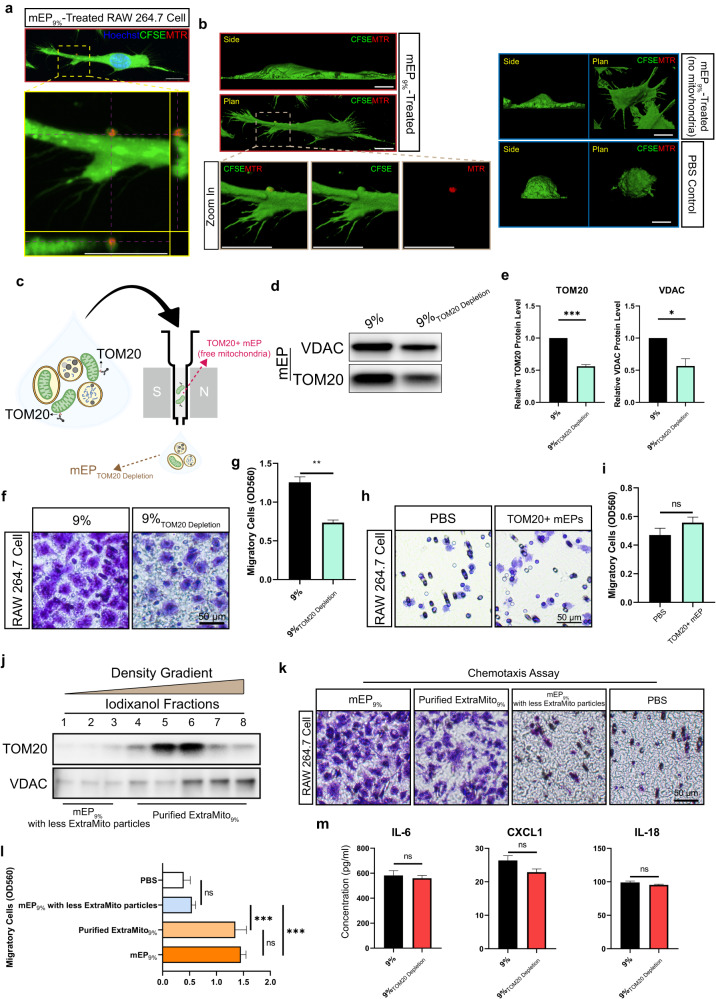
Tendon-derived ExtraMito particles, a subtype of mEP, are essential for inducing macrophage chemotaxis. **a** Representative 3D confocal images of RAW 264.7 cells during the uptake of MTR-labeled tendon-derived extracellular mitochondria, displayed as a maximum intensity projection (MIP). The lower panel shows orthogonal section views (x/y, x/z, or y/z) of the enlarged area marked by the yellow dashed box in the upper panel. Scale bar, 10 μm. **b** Representative side and plan view of 3D reconstruction rendering confocal images of RAW 264.7 cells uptaking MTR-labeled tendon-derived extracellular mitochondria (left, top and middle) and the corresponding enlargement of the area enclosed by the brown dashed box (left, bottom). The side and plan view of 3D reconstruction rendering confocal images of RAW 264.7 cells treated with mEP_9%_ but without MTR-labeled tendon-derived ExtraMito particles uptake observed (right, upper) and Raw 264.7 cells treated with the carrier (PBS) (right, bottom) are shown. Scale bar, 10 μm. **c** Schematic diagram of mitochondria depletion from tendon-derived mEPs by magnetic-activated cell sorting (MACS) technology. Free mitochondria were magnetically labeled with anti-TOM20 microbeads. Labeled TOM20+ mitochondria were retained in a paramagnetic column. **d** Immunoblot analysis of VDAC and TOM20 proteins in sham-depleted mEP_9%_ and TOM20-depleted mEP_9%_, and (**e**) the quantitative analysis of three biological replicates from three independent experiments. The mEPs that passed through paramagnetic columns without preincubation with TOM20 antibodies were sham-depleted mEPs. **f** Chemotaxis assay for RAW 264.7 cells toward sham-depleted mEP_9%_ or TOM20-depleted mEP_9%_, and (**g**) corresponding quantitative colorimetric measurements (*n* = 3 each group). Scale bar, 50 μm. **h** Chemotaxis assay for RAW 264.7 cell toward PBS or TOM20+ mEPs eluted from mEP_9%_ by MACS and (**i**) the corresponding quantitative colorimetric measurements (*n* = 3 per group). Scale bar, 50 μm. **j** After mEP_9%_ was separated through a high-resolution iodixanol density step gradient, representative immunoblot assays of different mEP_9%_ fractions of the mitochondrial markers TOM20 and VDAC demonstrated an enrichment of ExtraMito particles in Fractions 4–8; therefore, these fractions were defined as purified ExtraMito_9%_, and Fractions 1–3 were defined as mEP_9%_ with less ExtraMito particles. The same volume of each fraction (20 µL) was loaded into each lane. **k** Chemotaxis assay for RAW 264.7 cells toward unseparated mEP_9%_, purified ExtraMito_9%_, mEP_9%_ with less ExtraMito particles and PBS and (**l**) the corresponding quantitative colorimetric measurements (*n* = 3 each group). Scale bar, 50 μm. **m** Bead-based immunoassay showed that sham-depleted mEP_9%_ and TOM20-depleted mEP_9%_ induced macrophages to secrete cytokines. RAW 264.7 cells that migrated to the bottom of the insert microporous membrane in the Boyden chamber were stained. Quantitative colorimetric measurements are presented as three biological replicates from three independent experiments. The data are presented as the means ± SEMs. Student’s t test was used for statistical analysis. ***P < 0.001; **P < 0.01; *P < 0.05; ns, not significant.

To investigate whether the functional mEPs were directly absorbed by RAW 264.7 cells, we cultured RAW 264.7 cells with MTR-prelabeled tendon-derived ExtraMito particles within the mEP_9%_ population. After 24 h of incubation, we observed that the mitochondria from the tendon constructs could be taken up by RAW 264.7 cells (Fig. 6a). Interestingly, we noticed that the RAW 264.7 cells that absorbed the tendon-derived ExtraMito particles had a more elongated morphology than the cells not treated with mEP_9%_, and even compared to their counterparts treated with mEP_9%_ but without observable tendon-derived ExtraMito_9%_ uptake (Fig. 6b). Untreated RAW 264.7 cells displayed a rounded and plump morphology and lacked cytoplasmic extensions. In contrast, the RAW 264.7 cells treated with mEP_9%_ generally presented an activated phenotype with flattened and elongated shapes, which was more obvious for RAW 264.7 cells that absorbed the tendon-derived ExtraMito particles. These results suggested that the tendon-derived ExtraMito particles might exert specific effects on macrophages.

To the best of our knowledge, few studies have addressed the specific function of ExtraMito particles as a component of mEPs^42,43^. To determine the contribution of the tendon-derived ExtraMito particles within mEP_9%,_ which act as a chemoattractant to RAW 264.7 cells, we first depleted mitochondria from mEP_9%_ using the TOM20 antibody via MACS technology (Fig. 6c). Depletion of mitochondria was evaluated by the mitochondrial markers VDAC and TOM20 at the protein level (Fig. 6d, e). Immunoblotting revealed diminished levels of VDAC and TOM20 in TOM20-depleted mEP_9%_ compared to sham-depleted mEP_9%_. These results confirmed that post-MACS depletion, the mitochondrial component in mEPs, was strongly reduced. Immunoblotting also revealed residual ExtraMito particles in mEPs, corroborating the presence of mitochondria within particles resistant to MACS-mediated depletion. Next, TOM20-depleted mEP_9%_ or sham-depleted mEP_9%_ was added to a migration chamber system to serve as a chemoattractant. RAW 264.7 cells displayed a significantly reduced chemotaxis toward TOM20-depleted mEP_9%_ (Fig. 6f, g), compared to sham-depleted mEP_9%_, indicating the indispensability of ExtraMito particles within mEPs for macrophage chemotaxis. We then directly applied TOM20+ particles sorted from mEP_9%_ to RAW 264.7 cells as chemoattractants in the chemotaxis assay. Surprisingly, compared to those in response to PBS, TOM20+ particles did not significantly change RAW 264.7 cell chemotaxis (Fig. 6h, i), which implied that the ExtraMito particles of mEP_9%_ were necessary but not sufficient alone to induce macrophage chemotaxis.

To further confirm the capacity of mEP_9%_ ExtraMito particles to induce RAW 264.7 cell chemotaxis, we separated the mEP_9%_ fractions through a high-resolution iodixanol density step gradient (Supplementary Fig. 8). Immunoblotting of different mEP_9%_ fractions for the mitochondrial markers TOM20 and VDAC demonstrated enrichment of ExtraMito particles in Fractions 4–8 (Fig. 6j). We therefore defined Fractions 4–8 as purified ExtraMito_9%_ and Fractions 1–3 as mEP_9%_ with less ExtraMito particles. We subsequently introduced purified ExtraMito_9%_, mEP_9%_ with less ExtraMito particles, unseparated mEP_9%_, or PBS into the migration chamber system to serve as chemoattractants. Notably, purified ExtraMito_9%_ exhibited chemoattraction to RAW 264.7 cells that was comparable to that of unseparated mEP_9%_ (Fig. 6k, l). In contrast, the mEP_9%_ with less ExtraMito particles had negligible chemoattractive effects on RAW 264.7 cells, akin to the outcome of the PBS control group. These results highlight the important role of ExtraMito particles in inducing macrophage chemotaxis in a mechanical overload environment.

Next, to further explore the role of ExtraMito particles, a component of mEP_9%_ that induces inflammatory cytokine release from macrophages, RAW 264.7 cells were incubated with TOM20-depleted mEP_9%_ or sham-depleted mEP_9%_. The bead-based multiplex assay revealed that there were no significant changes in the release of IL-6, CXCL1, or IL-18 after the depletion of ExtraMito particles from mEPs (Fig. 6m), implying that the rest components of mEP_9%,_ except for mitochondria, mainly induced inflammation. Taken together, these data suggest that ExtraMito particles, as a component of mEPs, mainly contributes to macrophage chemotaxis.

### Protection of the intracellular mitochondrial network in overloaded parental tendon cells prevents macrophage chemotaxis induced by ExtraMito particles

**Fig. 7:**
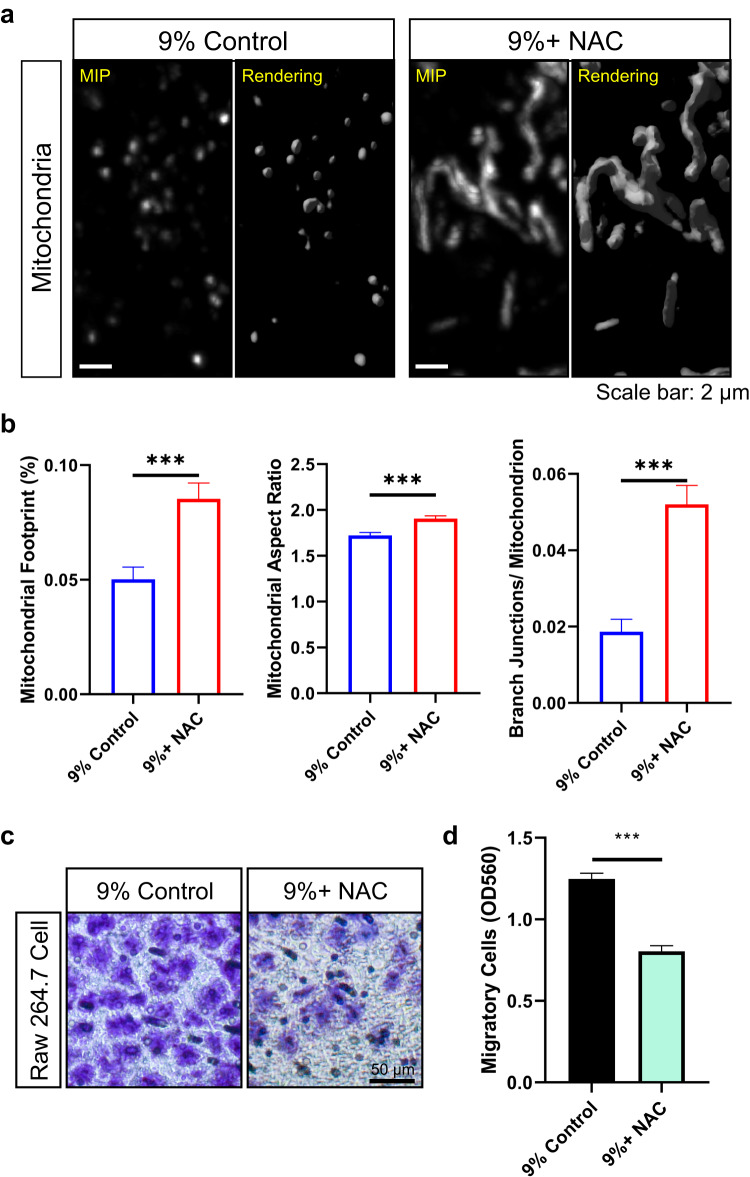
Protection of the intracellular mitochondrial network in overloaded tendon cells prevents macrophage chemotaxis induced by ExtraMito particles. **a** Representative 3D live-cell confocal images of MTR-labeled mitochondria in tendon constructs after 9% strain supplied with or without N-acetylcysteine (NAC), shown as MIP and 3D reconstruction rendering images (scale bar, 2 μm); (**b**) corresponding quantification of mitochondrial morphology analysis (*n* = 15 each group); (**c**) chemotaxis assay for RAW 264.7 cells toward mEPs from 3D tendon constructs after 9% strain supplied with or without NAC (scale bar, 50 μm); and (**d**) corresponding quantitative colorimetric measurement. Quantitative colorimetric measurements are presented as three biological replicates from three independent experiments. The data are presented as the means ± SEMs. Student’s t test was used for statistical analysis. ***P < 0.001; MIP maximum intensity projection.

Given that overload impairs the intracellular mitochondrial network, we hypothesized that the damaged intracellular mitochondrial network causes the release of ExtraMito particles, which can induce macrophage chemotaxis. We subsequently applied N-acetylcysteine (NAC), an antioxidant normally supplied as a mitochondrial protector and stressed mitochondria rescuer^44,45^, to regulate mitochondrial homeostasis in tendon constructs in a mechanical overload environment with 9% strain. Live-cell CLSM showed that NAC treatment could reverse the impairment of the mitochondrial network labelled by MTR in tendon constructs receiving 9% strain (Fig. 7a). Quantitative analysis revealed a larger mitochondrial footprint, a greater mitochondrial aspect ratio, and more branch junctions per mitochondrion in the tendon constructs after NAC treatment in the 9% strain environment (Fig. 7b). Then, the mEPs released by the tendon constructs subjected to 9% strain and treated with NAC (termed NAC-treated mEP_9%_) were collected and applied to a migration chamber system as a chemoattractant for RAW 264.7 cells. As expected, the chemotaxis assay demonstrated that, compared to mEP_9%_, NAC-treated mEP_9%_ significantly lost the capacity to induce macrophage chemotaxis (Fig. 7c, d), indicating that the impaired intracellular mitochondrial network was responsible for macrophage chemotaxis induced by mEP_9%_.

## Discussion

**Fig. 8:**
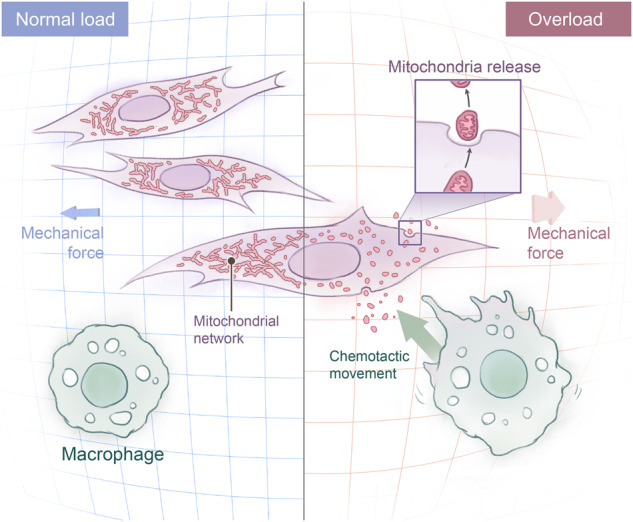
A schematic diagram showing that ExtraMito particles act as cell signal that induces inflammation in overload-induced tendinopathy. Optimal load activates the mitochondrial network, while overload damages the mitochondrial network in tendon cells. Furthermore, overload-impaired mitochondria can be secreted into the extracellular environment, which then induces macrophage chemotaxis toward the damaged sites, resulting in inflammation.

In the present study, we showed that the release of ExtraMito particles from tendon cells during mechanical overload is a novel pathological mechanism of tendinopathy. We showed that these newly identified tendon-derived ExtraMito particles serve as a cell‒cell communicators to transmit mechanical signals from mechanically stimulated tendon cells to macrophages (Fig. 8). Our data indicate that overload damages the intracellular mitochondrial network and triggers the release of ExtraMito particles and mEPs by tendon cells. Then, the ExtraMito particles and mEPs promote the chemotaxis of macrophages and the release of inflammatory cytokines, ultimately causing inflammatory reactions. By protecting the intracellular mitochondrial network in tendon cells from breakdown, we can reduce overload-induced macrophage chemotaxis.

Inflammation is considered a contributing factor to tendinopathy. Immune cell infiltration and further cytokine production are essential for inflammatory reactions. Our work indicated that both of these processes were dependent on the degree of mechanical stimulation, supporting the theory that overload initiates the pathological progression of tendinopathy^2,8^. Macrophage infiltration has been characterized in human tendinopathy^46^. The present study revealed that the ExtraMito particles within mEPs are fundamental for inducing macrophage infiltration. Several proinflammatory cytokines, such as IL-1β, TNF-α, CXCL1 and IL-6, have been reported to initiate catabolic processes, especially in the early phase of tendinopathy^13,47,48^. We found that the mechanism underlying the loading-induced release of IL-6, CXCL1, and IL-18 was dependent on the degree of mechanical loading. Among them, IL-6 and CXCL1 secretion was associated with mEPs released from tendon cells in an overload environment. These data suggest that the newly identified tendon-derived mEPs and ExtraMito particles affect the immune microenvironment from several perspectives, including both macrophage infiltration and cytokine production.

The bioreactor platform we applied in the present study was a 3D uniaxial model. Compared to a 2D loading model, this 3D loading model allows tendon cells to receive mechanical stimuli not only through cell‒cell interactions but also via cell–ECM interactions across the entire cell body. Previous work has suggested that this platform can more closely mimic the in vivo tendon mechanical environment for tendon cells from the perspective of inducing differentiation^25^. The present study further proved that it could induce tendon cells to secrete extracellular particles. These extracellular factors can ultimately affect macrophages to create an immune microenvironment similar to that in vivo.

Using live-cell confocal microscopy, we observed that mitochondria were responsive to mechanical loading. Mitochondria, the “energy power stations” of cells, can transform between elongated or fragmented states and into interconnected networks to maintain their fitness, which is required for normal cell metabolism^49,50^. An interconnected mitochondrial network is a combination of diverse mitochondrial morphologies indicating active mitochondrial dynamics, which increases the controllability, robustness, and efficiency of the network in response to perturbations, as well as enhancing oxidative capacity^34,35^. These networks appeared more frequently when the cells were in a normal loading environment in the present study. Consistent with the findings of previous studies^26,51,52^, the present study confirmed through the analysis of mitochondrial geometric features that only moderate mechanical loading is beneficial for cell metabolism.

Mitochondria-targeted drugs have been applied in animal studies and clinical trials for several diseases, such as Parkinson’s disease, cardiovascular disease, and cancer^53,54^. Although oxidative stress has been reported to be involved in tendinopathy^55^, to the best of our knowledge, no mitochondria-targeted drug has been reported to be clinically used for treating tendinopathy. The present study showed that protection of mitochondria from overload not only rescues tendon cells themselves but also improves the immune microenvironment, which highlights the potential of mitochondria treatment for tendinopathy.

The nomenclature used in the field of extracellular particles/extracellular vesicles is rapidly evolving. Microvesicles are heterogeneous extracellular vesicles that typically range in size from 150 nm to 1000 nm. However, in some cases, they can be as large as 10 μm^56,57^. Some studies have termed mitochondria-derived extracellular vesicles “mitovesicles“^43,58^. Some studies have referred to these vesicles as “mitochondria extracellular vesicles” or “extracellular mitochondria“^29,59^. The term “microvesicle” has been assigned to an extracellular vesicle in a plasma membrane-derived biogenesis pathway^60^, which was not discussed in the present study. Thus, in this study, we opted for a more general term, “mEPs,” to describe these medium-sized particles/vesicles, and we used “ExtraMito” to denote the mitochondrial portion. Previous studies have shown that these particles, which can be released from activated monocytes or platelets, are related to inflammatory reactions^29,36,61^. The limitation of some previous studies is that their extracellular particles were heterogeneous, meaning that the function of the mitochondrial portion was not distinguished from that of the nonmitochondrial part^42,43^. Using the Migration Chamber system and MACS technology, we showed for the first time that the ExtraMito portion of mEPs from overloaded tendon constructs is indispensable for macrophage chemotaxis. Interestingly, our data showed that the mitochondrial portion could not work alone as a chemoattractant. However, the lack of these mitochondria particles significantly reduced macrophage chemotaxis. As chemotaxis requires three independent steps—chemosensing, polarization and locomotion^62^—we hypothesized that ExtraMito particles participate in only a part of these steps. Since we observed morphological changes in the polarization of RAW 264.7 cells after the uptake of tendon-derived ExtraMito particles, even when compared to their counterparts treated with mEPs but without ExtraMito particles uptake, we assumed that ExtraMito particles mainly worked through the polarization step. As we did not obtain the mitochondrial portion encapsulated in mEPs, synergy between the free mitochondrial portion and the mitochondrial portion encapsulated in mEPs could not be inferred. Tools to extract the specific particle portions are lacking and need to be developed.

The field of mechanobiology is interdisciplinary and aims to study how force propagates in cells and affects cells and how cells, in turn, remodel the microenvironment to adapt to physical and mechanical stimuli. While studies in this field have traditionally focused on individual cell types, cell–cell communication is a critical process for maintaining biological homeostasis. This work represents the first demonstration of ExtraMito particles functioning as an intercellular crosstalk signal in mechanobiology. While our conclusions were drawn based on tendinopathy, these findings may extend to other diseases induced by mechanical overload, such as pressure overload-induced cardiac fibrosis and heart failure^63^ and ocular hypertension-caused glaucoma^64^, as inflammation has been found to be an essential associated event in these overload-induced diseases^65,66^.

In conclusion, we provide evidence that tendon cells can release mEPs into the extracellular environment, which contain mechanical response messages that regulate the immune microenvironment. Furthermore, the ExtraMito portion of mEPs is essential for inducing macrophage chemotaxis. Our data reveal a novel pathological mechanism of tendinopathy and provide new insights for the treatment of tendinopathy and related inflammatory reactions.

### Supplementary information

Supplementary Information
