# Supplementary material for: Mechanical overload-induced release of extracellular mitochondrial particles from tendon cells leads to inflammation in tendinopathy
Source: Exp Mol Med. 2024 Mar 1;56(3):583–99. doi: 10.1038/s12276-024-01183-5 (PMC10985099; doi:10.1038/s12276-024-01183-5)
Supplement: Supplementary file 1 — Supplementary Information [file 12276_2024_1183_MOESM1_ESM.pdf]

Supplementary Information

Supplementary Fig. 1. Microarray data reveal that oxygen-related reactions, extracellular particles, and inflammation are activated in human tendinopathy

Supplementary Fig. 2. Finite Element Analysis (FEA) shows strain distribution in samples prepared for microscopy and immunoblot experiments

Supplementary Fig. 3. Size characterization of medium extracellular particles (mEPs) by flow cytometry

Supplementary Fig. 4. Representative immunoblot analyses of protein markers for mEPs

Supplementary Fig. 5. Two forms of extracellular mitochondria (ExtraMito) are identified

Supplementary Fig. 6. Bead-based immunoassay for cytokines detection to mEPs

Supplementary Fig. 7. Bead-based immunoassay shows that mEPs mediate RAW 264.7 cells to release various cytokines in a dose-dependent manner

Supplementary Fig. 8. Schematic protocol for separating mEPs fractions by high-resolution iodixanol density gradient

Supplementary Fig. 9. Uncropped images of immunoblot analyses

Supplementary Table 1. Top 50 up-regulated and down-regulated genes in human tendons with tendinopathy compared to healthy human tendons

Supplementary Table 2. Primer sequences for quantitative PCR (qPCR)

Supplementary Fig. 1.

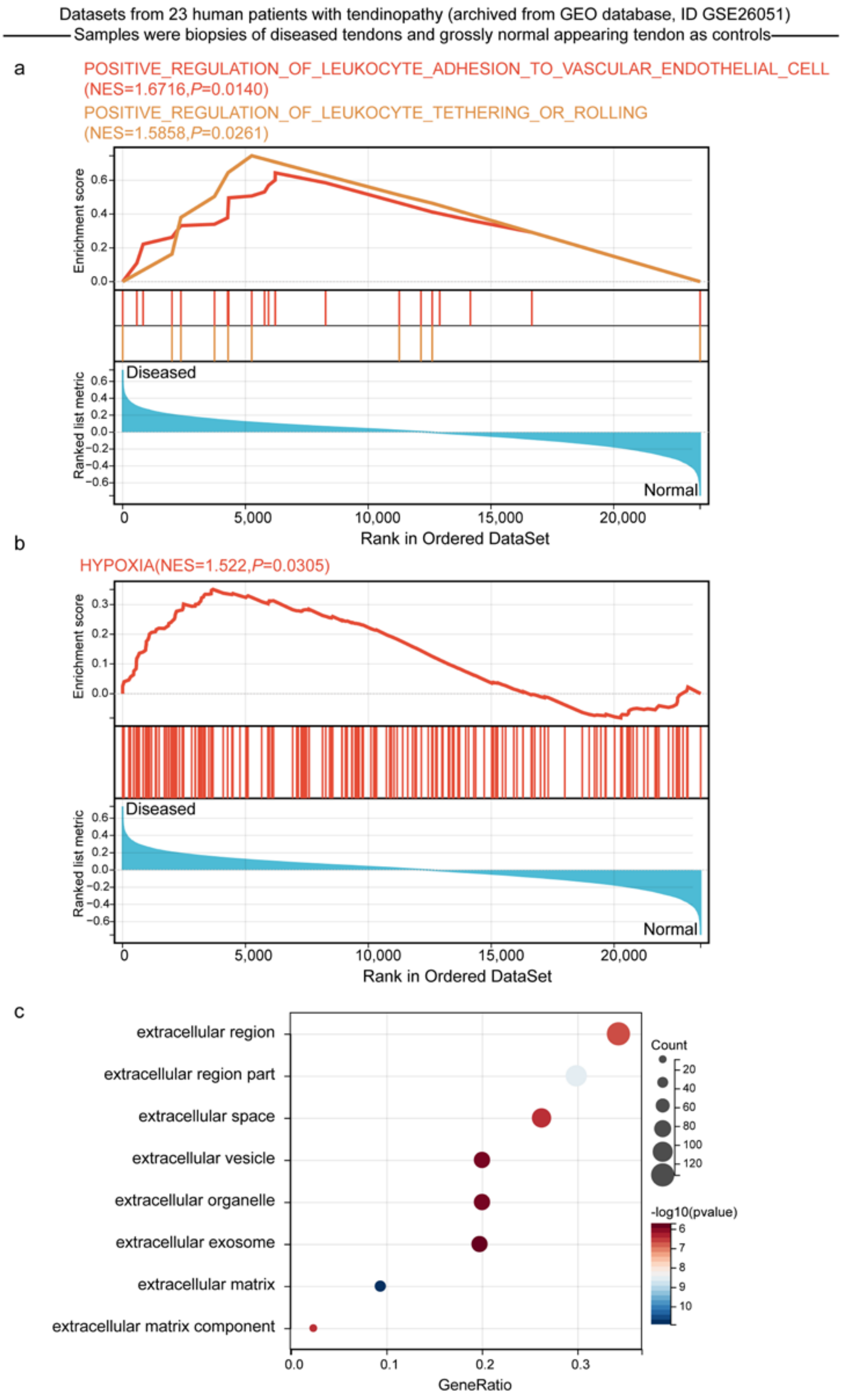

Supplementary Fig. 1. Microarray data reveal that oxygen-related reactions, extracellular particles, and inflammation are activated in human tendinopathy (a) Gene Set Enrichment Analysis (GSEA) plots for “positive regulation of leukocyte

adhesion of vascular endothelial cell" gene set, and "positive regulation of leukocyte tethering or rolling" gene set from GO terms for BP showing significantly activated in human tendinopathy with both $P < 0.05$. (**b**) GSEA plots for "Hypoxia" gene set from hallmark gene sets showing positively associated with tendinopathy ($P = 0.0305$). (**c**) Gene ontology cellular component analysis of the top 500 up-regulated genes in tendons with tendinopathy compared to healthy tendons showed that most activated genes in tendinopathy were significantly related to extracellular region and extracellular particles (all $P < 0.05$). GEO, gene expression omnibus; NES, normalized enrichment score.

Supplementary Fig. 2

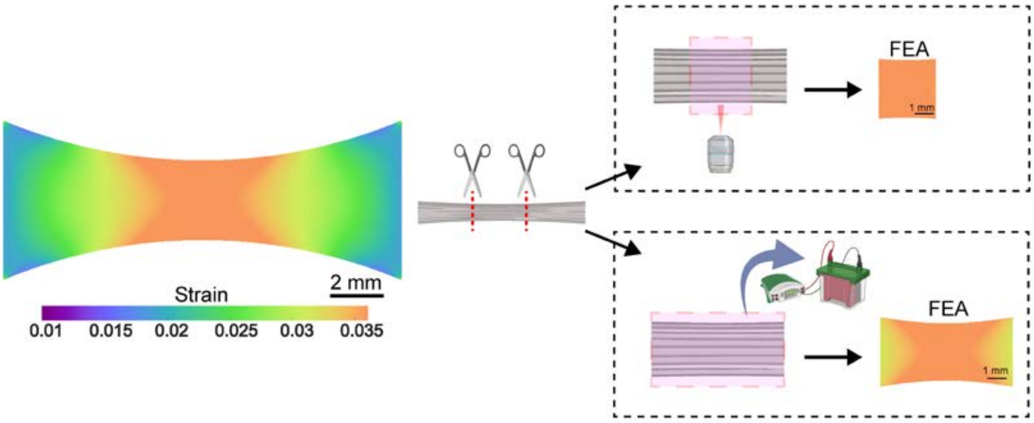

Supplementary Fig. 2. Finite Element Analysis (FEA) shows strain distribution in samples prepared for microscopy and immunoblot experiments Narrower sample sections were used for microscopy experiments (right upper panel) than immunoblot experiments (right bottom panel), resulting in higher mean strain.

Supplementary Fig. 3

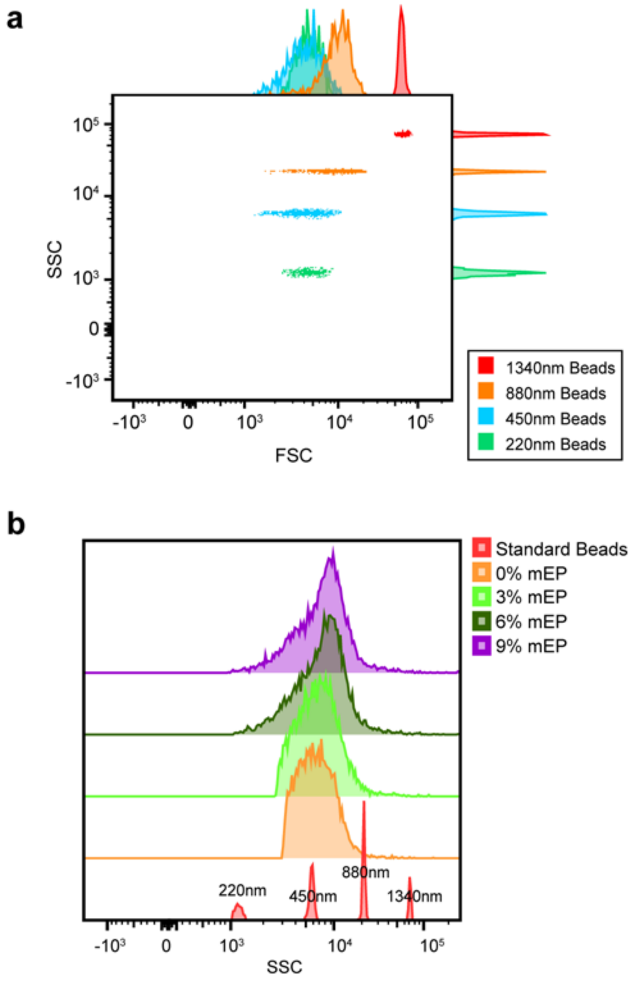

Supplementary Fig. 3. Size characterization of medium extracellular particles (mEPs) by flow cytometry (**a**) Gating strategy for mEPs characterization and quantification by flow cytometry. The detection gate was defined by standard nanobeads sized 220nm (Green), 450nm (Blue), 880nm (Orange), and 1340nm (Red). (**b**) Histogram of the size of mEPs from tendon constructs receiving 0%, 3%, 6%, or 9% cyclic strain, characterized by flow cytometry.

Supplementary Fig. 4

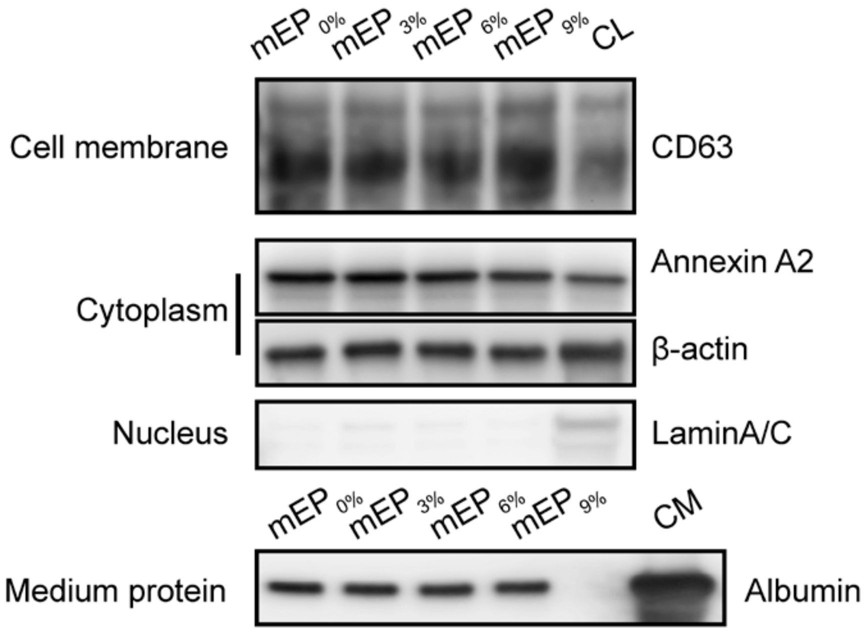

Supplementary Fig. 4. Representative immunoblot analyses of protein markers for mEPs CL, cell lysis; CM, culture medium (10 μL of CM was loaded).

Supplementary Fig. 5

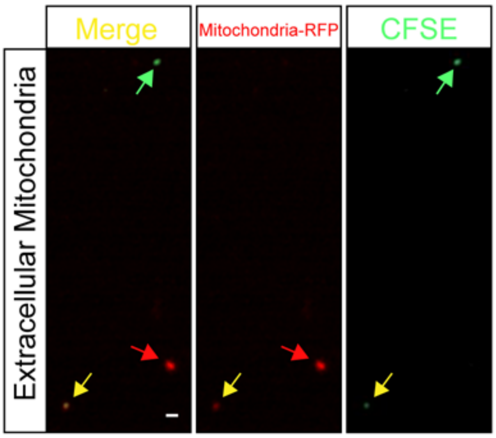

Supplementary Fig. 5. Two forms of extracellular mitochondria (ExtraMito) are identified Mitochondria were labeled by transfection with a red fluorescence protein (RFP) fused to mitochondria, and cytoplasm was stained with carboxyfluorescein succinimidyl ester (CFSE) in tendon constructs before applying mechanical stimulations. Confocal fluorescence microscopy showed two forms of ExtraMito, including mitochondria (Red) encapsulated in mEPs (Green) and free extracellular mitochondria (Red). Scale bar, 1 μm.

Supplementary Fig. 6

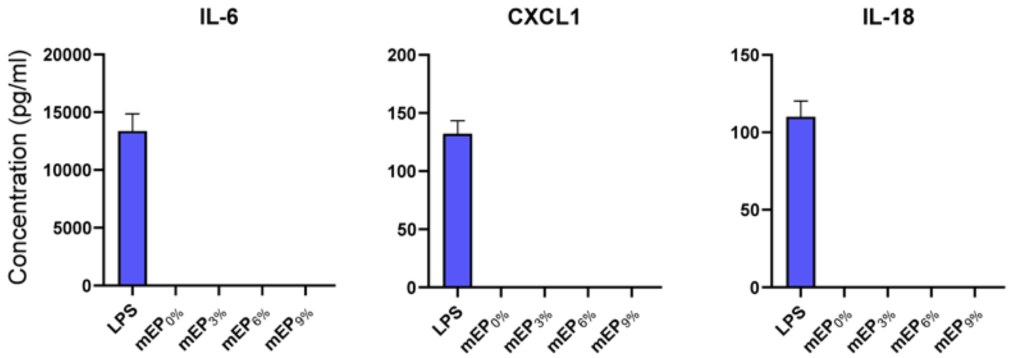

Supplementary Fig. 6. Bead-based immunoassay for cytokines detection to mEPs Bead-based immunoassay showed that IL-6, CXCL1 and IL-18 inside the mEPs were below the detectable threshold (n=3 each group). 500 ng/ml Lipopolysaccharides (LPS) was used as the positive control to induce cytokines release from RAW 264.7 cells.

Supplementary Fig. 7

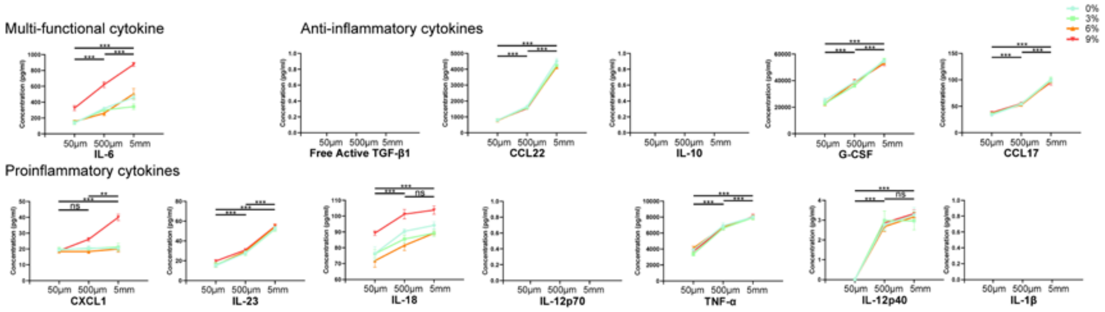

Supplementary Fig. 7. Bead-based immunoassay shows that mEPs mediate RAW 264.7 cells to release various cytokines in a dose-dependent manner Bead-based immunoassay showed that the mEPs mediated RAW 264.7 cells to release IL-6, CCL22, G-CSF, CCL17, CXCL1, IL-23, IL-18, TNF-α, and IL-12p40 in a dose-dependent manner (n=3 each group). The concentration of mEPs was set using a 10-fold gradient. This included mEPs from tendon constructs of 50 μm, 500 μm, and 5 mm. Data presented as means ± SEM. Two-way ANOVA with Tukey's multiple comparisons test within different doses for main concentration effect statistical analysis. ***$P < 0.001$; **$P < 0.01$; ns, not significant.

Supplementary Fig. 8

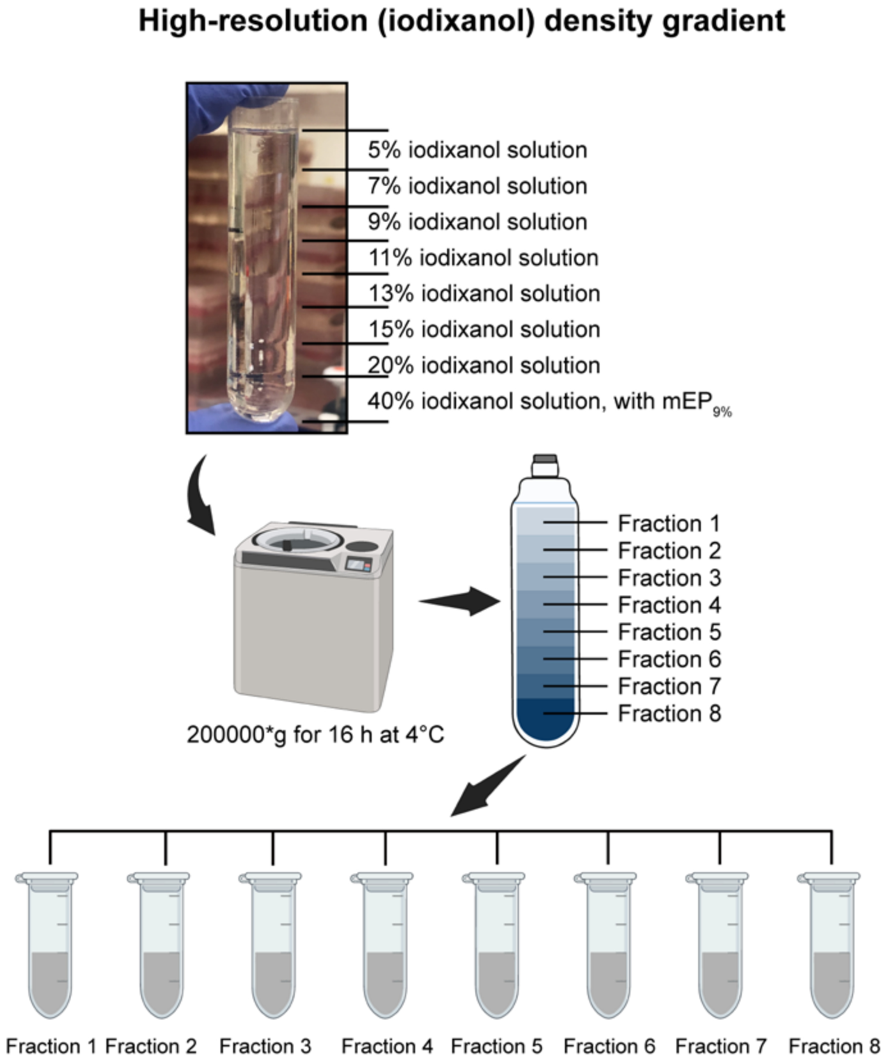

Supplementary Fig. 8. Schematic protocol for separating mEPs fractions by high-resolution iodixanol density gradient The pellet containing medium extracellular particles (mEPs) was resuspended in a 40% (v/v) iodixanol (OptiPrep) solution. A density step gradient was set up by carefully layering decreasing concentrations of iodixanol (20, 15, 13, 11, 9, 7, and 5%) on the top of the 40% iodixanol solution containing the mEPs. The column was then centrifuged for 16 hours at 4 °C at 200,000 g. Afterward, 1.25 mL of the gradient from the top (Fraction 1) was collected, and 1.5 mL fractions (Fraction 2-8) were subsequently collected. Each fraction was mixed with 4 mL ice-cold phosphate buffered saline (PBS) except that Fraction 8 was mixed with 8 mL ice-cold PBS. Fractions were then centrifuged at 18000 g for 30 min to collect the pellets of each fraction.

Supplementary Fig. 9

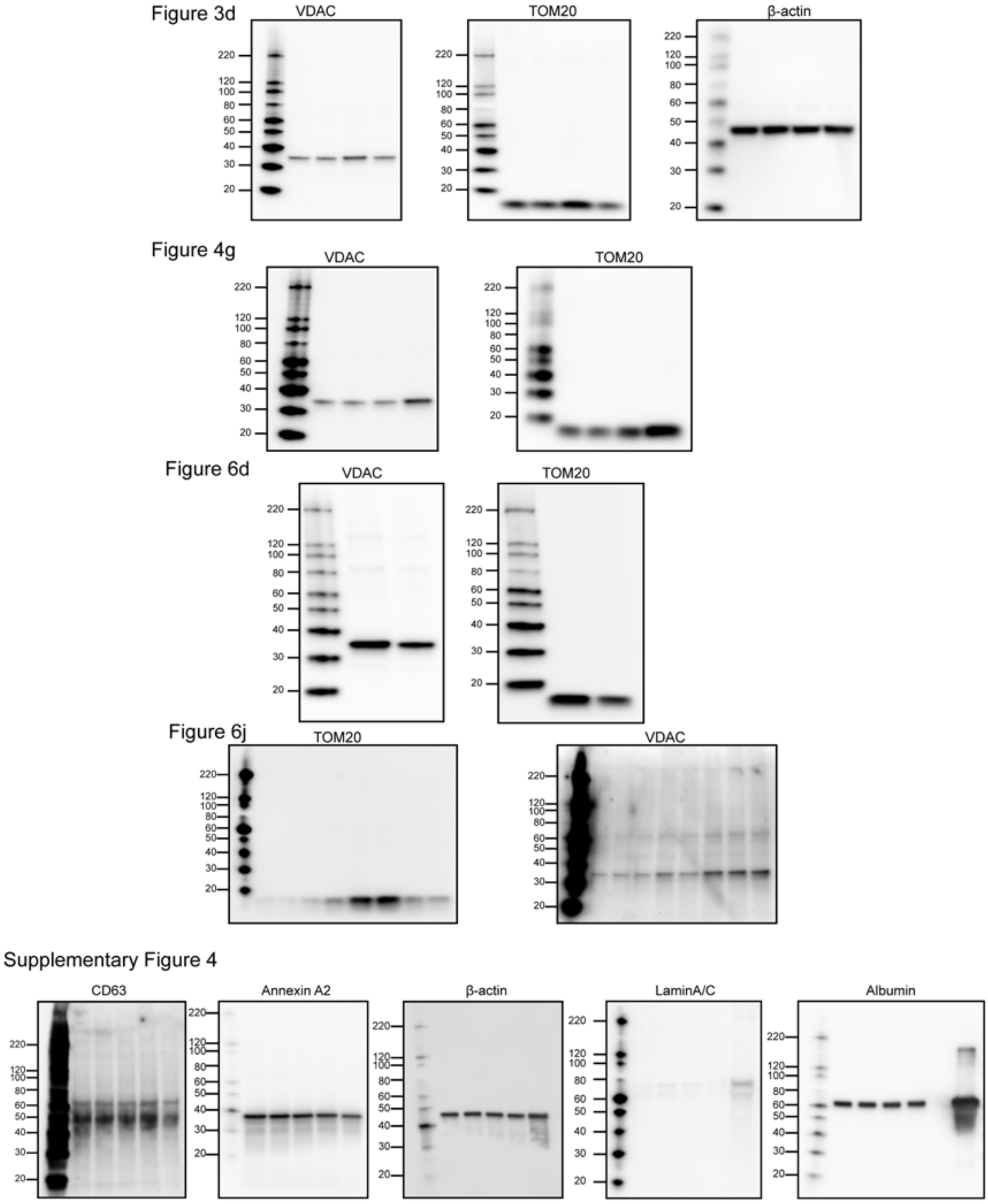

Supplementary Fig. 9. Uncropped images of immunoblot analyses

Supplementary Table 1. Top 50 up-regulated and down-regulated genes in human tendons with tendinopathy compared to healthy human tendons

AccID	log2FC
ENSG00000148671	25.28443
ENSG00000007908	12.08962
ENSG00000229807	11.41345
ENSG00000064886	10.07536
ENSG00000154319	9.916218
ENSG00000214688	9.756219
ENSG00000131668	9.59858
ENSG00000101158	9.213841
ENSG00000159167	8.967327
ENSG00000138795	8.859301
ENSG00000142694	8.787869
ENSG00000105509	8.662856
ENSG00000210195	8.564318
ENSG00000178922	8.509227
ENSG00000168282	8.491517
ENSG00000100985	8.445448
ENSG00000160818	8.439592
ENSG00000164708	8.372936
ENSG00000230043	8.058353
ENSG00000165474	8.010397
ENSG00000109158	7.968655
ENSG00000225383	7.962709
ENSG00000152931	7.913135
ENSG00000173452	7.903374
ENSG00000017483	7.897234
ENSG00000124731	7.874496
ENSG00000174640	7.865826
ENSG00000095713	7.83314
ENSG00000249306	7.777503
ENSG00000124479	7.656688
ENSG00000173349	7.64469
ENSG00000273149	7.639296
ENSG00000102931	7.582288
ENSG00000230838	7.575987
ENSG00000064195	7.574989
ENSG00000157551	7.569256
ENSG00000210151	7.567284
ENSG00000138944	7.405685
ENSG00000134668	7.388755
ENSG00000157542	7.369604
ENSG00000241717	7.303429
ENSG00000047457	7.27848

ENSG00000144057	7.268987
ENSG00000204866	7.253752
ENSG00000179292	7.239306
ENSG00000136305	7.236785
ENSG00000136867	7.096525
ENSG00000103316	7.093536
ENSG00000261327	7.071528
ENSG00000234961	7.054262
ENSG00000229246	-11.548
ENSG00000185960	-10.8953
ENSG00000148677	-10.6316
ENSG00000284633	-10.5888
ENSG00000133055	-10.2487
ENSG00000258231	-10.2329
ENSG00000253500	-10.169
ENSG00000198857	-10.1487
ENSG00000199295	-9.99573
ENSG00000225613	-9.94849
ENSG00000214281	-9.71798
ENSG00000188729	-9.67939
ENSG00000136928	-9.6752
ENSG00000286884	-9.60681
ENSG00000096264	-9.55608
ENSG00000215182	-9.46777
ENSG00000287831	-9.46112
ENSG00000159251	-9.4287
ENSG00000257243	-9.42012
ENSG00000167768	-9.37501
ENSG00000121207	-9.35968
ENSG00000187550	-9.34396
ENSG00000133020	-9.29746
ENSG00000223511	-9.27306
ENSG00000239570	-9.27306
ENSG00000041515	-9.24263
ENSG00000169989	-9.22063
ENSG00000288094	-9.21999
ENSG00000226620	-9.20922
ENSG00000234132	-9.19517
ENSG00000236858	-9.16489
ENSG00000238078	-9.16136
ENSG00000234005	-9.04795
ENSG00000240021	-9.04795
ENSG00000287373	-8.98642
ENSG00000112041	-8.98572
ENSG00000233393	-8.98572
ENSG00000252264	-8.98572
ENSG00000174279	-8.97097

ENSG00000168878	-8.93707
ENSG00000147160	-8.9207
ENSG00000285939	-8.9207
ENSG00000120057	-8.90558
ENSG00000226087	-8.89336
ENSG00000282915	-8.85261
ENSG00000237560	-8.82328
ENSG00000139330	-8.80638
ENSG00000253660	-8.78114
ENSG00000253227	-8.76456
ENSG00000232258	-8.71637

Supplementary Table 2. Primer sequences for quantitative PCR (qPCR)

Gene	Primer sequence	
	Forward 5’->3’	Reverse 5’->3’
COL1A1	TGACTGGAAGAGCGGAGAGT	GTTCGGGCTGATGTACCAGT
Scleraxis	CCCAAACAGATCTGCACCTT	GGCTCTCCGTGACTCTTCAG
Mohawk	GTCCGGCAGCCAGATTTAAG	TCGCTGAGCTTTCCCCTTTA
Tenomodulin	CCGCAGAAAAGCCTATTGAA	GACCACCCATTGCTCATTCT
36B4	CTTCCCACTTGCTGAAAAGG	CGAAGAGACCGAATCCCATA
